# IGF2BP3-dependent glutamine/BCAA metabolic rewiring rejuvenates aged human adipose-derived stem cells for enhanced tissue regeneration

**DOI:** 10.1038/s41421-025-00860-7

**Published:** 2026-01-20

**Authors:** Zichao Li, Lin Feng, Xinxin Wei, Huichen Li, Yifu Zhu, Hongtao Wang, Jiaqi Liu, Liang Luo, Zhao Zheng, Baoqiang Song, Liangliang Shen, Dahai Hu

**Affiliations:** 1 Department of Burns and Cutaneous Surgery, Xijing Hospital, Fourth Military Medical University, Xi’an, Shaanxi China; 2 Department of Plastic and Aesthetic Surgery, Peking Union Medical College Hospital, Chinese Academy of Medical Sciences & Peking Union Medical College, Beijing, China; 3 Department of Plastic Surgery, Xijing Hospital, Fourth Military Medical University, Xi’an, Shaanxi China; 4 Department of Basic Medicine, Xi’an Medical University, Xi’an, Shaanxi China; 5 Department of Biochemistry and Molecular Biology, Fourth Military Medical University, Xi’an, Shaanxi China; 6 Department of Stomatology, Weifang People’s Hospital, Weifang, Shandong China; 7 State Key Laboratory of Holistic Integrative Management of Gastrointestinal Cancers and National Clinical Research Center for Digestive Diseases, Fourth Military Medical University, Xi’an, Shaanxi China

**Keywords:** Ageing, Reprogramming, Mesenchymal stem cells

## Abstract

Aging impairs the regenerative capacity and differentiation potential of human adipose-derived stem cells (hASCs), but the mechanisms underlying their functional decline remain unclear. Through systematic functional assays and in vivo experiments, we first confirmed age-associated reductions in hASC self-renewal, lineage plasticity, and tissue repair efficacy. By integrating multiomics profiling and functional validation, we identified a metabolically active ACTA2^+^TAGLN^+^ subpopulation that was enriched mainly in infant-derived hASCs (I-hASCs) and characterized by increased catabolism of branched-chain amino acids (BCAAs) and glutamine. Mechanistically, the RNA-binding protein IGF2BP3, which is predominantly expressed in the ACTA2^+^TAGLN^+^ subpopulation, sustains hASC stemness by stabilizing BCAT1 and GLS mRNAs via METTL3-mediated m6A modification, thereby preserving redox homeostasis and mitochondrial energy production. Furthermore, age-related attenuation of the IGF2BP3-m6A-BCAT1/GLS axis contributed to metabolic reprogramming, driving senescence-associated functional collapse in elderly-derived hASCs (E-hASCs). Strikingly, rescue experiments demonstrated that genetic restoration of BCAT1/GLS or supplementation with BCAAs/glutamine significantly rejuvenated E-hASCs, restoring their proliferation, differentiation, and in vivo wound-healing capacities. These findings identify IGF2BP3 as a central regulator of hASC aging by linking m6A epitranscriptomic modifications to metabolic reprogramming and establish the IGF2BP3-m6A-BCAT1/GLS axis as a druggable node in aged hASCs. This study proposed two therapeutic strategies: nutrient supplementation to rescue metabolic deficits and m6A modulation to stabilize key mRNAs, providing a clinically feasible protocol to optimize elderly-derived hASCs for tissue regeneration.

## Introduction

Human mesenchymal stem cells (hMSCs) play crucial roles in organ development, tissue homeostasis, repair, and reconstruction^1^. Human adipose-derived stem cells (hASCs) are mesenchymal stem cells in adipose tissue with self-renewal and multipotent differentiation capacities. The use of hASCs and their derivatives is a desirable approach in cell-assisted therapy (CAT), offering promising prospects for tissue repair and the treatment of degenerative conditions and metabolic diseases^2–4^. However, age-related changes have been observed in stem cells obtained from older donors, and these cells disrupt tissue homeostasis and contribute to the onset of aging-related diseases^5^. Research on the age-related characteristics of hASCs has demonstrated a decrease in genome integrity, reduced self-renewal potential, diminished therapeutic efficacy, and even accelerated aging in elderly individuals^6,7^. Consequently, addressing age-related cellular senescence in hASCs is crucial for enhancing their clinical utility in elderly patients.

Aging is a complex and unavoidable process characterized by age-related metabolic dysfunction in MSCs, which substantially compromises their biological properties and induces senescence^8,9^. Metabolites are crucial for influencing lifespan and the aging process^10^. Key metabolic pathways, including the tricarboxylic acid (TCA) cycle, branched-chain amino acid (BCAA) metabolism, lipid metabolism, and nicotinamide adenine dinucleotide (NAD^+^) metabolism, are associated with cellular senescence and organismal aging^11–13^. Glutathione (GSH) is an intracellular antioxidant that neutralizes reactive oxygen species (ROS) to maintain cellular homeostasis and delay cellular senescence^14^. Reduced GSH levels have been linked to senescence in hMSCs^15,16^. Accordingly, GSH has emerged as a critical component in aging-associated metabolic pathways, offering potential avenues for reversing cellular senescence, including in hMSCs. However, the effects of nutrients on aging-related diseases remain debated^17,18^. Additional research is needed to comprehensively address the intricate interplay between nutrient metabolism and aging.

N6-Methyladenosine (m6A), the most prevalent reversible post-transcriptional RNA modification in eukaryotes, is dynamically regulated by a methylase system consisting of methyltransferases (“writers”), demethylases (“erasers”), and binding proteins (“readers”)^19,20^. Emerging evidence suggests that m6A modification is intricately linked to cell fate decisions, stemness maintenance, and senescence progression in hMSCs^21^. Recently, studies have demonstrated that m6A-mediated regulation extends to the lineage plasticity of human adipose-derived stem cells (hASCs) by modulating osteogenic and adipogenic differentiation programs^22,23^. Notably, mechanistic studies have revealed that m6A-dependent regulation of GSH metabolism may regulate redox homeostasis and cellular senescence through ROS-mediated signaling pathways^24,25^. However, the pivotal question remains unresolved: do m6A modifications affect aging-associated metabolic reprogramming, ultimately dictating the senescence trajectory in hMSCs?

Single-cell RNA sequencing (scRNA-seq) has revealed intrapopulation heterogeneity of hASCs during differentiation^26,27^. Aging disrupts the metabolic and epigenetic features critical for the cellular components and stemness of hASCs. However, the molecular nodes coordinating these alterations remain unknown. In this study, we used multiomics profiling of infant- and elderly-derived hASCs to reveal age-dependent metabolic rewiring, specifically identifying insulin-like growth factor 2 mRNA-binding protein 3 (IGF2BP3) as an m6A-dependent stabilizer of branched-chain amino acid transaminase 1 (BCAT1) and glutaminase (GLS) mRNAs, which encode key regulators of BCAA and glutamine metabolism. We demonstrate that epigenetic–metabolic interplay drives hASCs aging via orthogonal validation spanning in vitro cell stemness analyses, BCAA/glutamine metabolism assessments, and in vivo wound healing/fat graft systems. Genetic and metabolic interventions establish causality between m6A epitranscriptomics and nutrient-sensitive pathways, whereas single-cell mapping reveals dynamic intercellular networks that amplify these defects in aged hASCs. This study elucidates the role of the IGF2BP3-BCAT1/GLS axis as an epigenetic–metabolic regulator of stemness and provides a dual-action strategy — combining m6A modulation and nutrient supplementation — to rejuvenate aged hASCs. These insights directly address the clinical bottleneck of autologous stem cell therapies in elderly individuals, offering scalable solutions to enhance tissue repair, combat degenerative diseases, and improve regenerative outcomes across age-related pathologies.

## Results

### The self-renewal and multidirectional differentiation potential of hASCs decrease with age

hASCs are widely used in the clinic primarily because of their high activity, which enables their self-renewal and multidirectional differentiation potential in vivo and in vitro, ultimately improving tissue repair and the management of degenerative disorders^28^. However, it is crucial to acknowledge that an age-related decline in cell activity can compromise the therapeutic efficacy of autologous stem cell transplantation^29^.

**Fig. 1:**
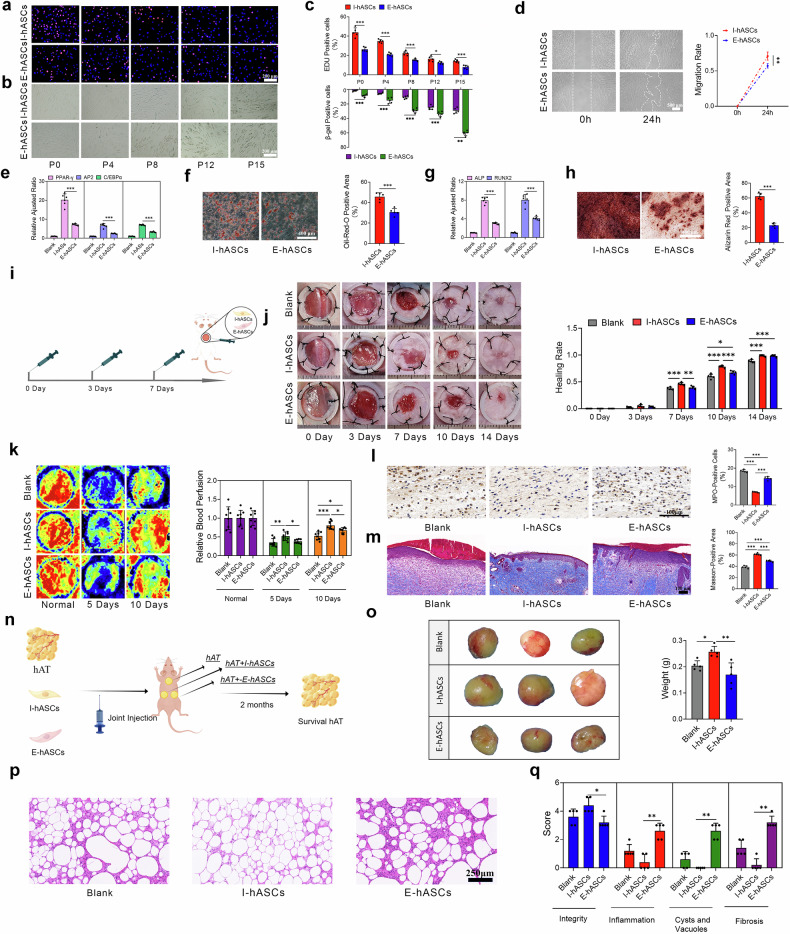
Age-associated decreases in self-renewal capacity, lineage plasticity, and tissue remodeling efficacy in hASCs. **a** Representative images of EdU staining of I-hASCs and E-hASCs cultured at passages 0, 4, 8, 12, and 15. Red represents EdU-positive cells. **b** SA-β-gal staining of I-hASCs and E-hASCs cultured at passages 0, 4, 8, 12, and 15. Green indicates SA-β-gal-positive cells. **c** Quantification of EdU- and SA-β-Gal-positive cell percentages at different passages in vitro. **d** Scratch assay results and quantification of the migration rates of primary I-hASCs and E-hASCs after 24 h of culture. **e** Relative expression of lipogenic genes in primary I-hASCs and E-hASCs after three weeks of adipogenic induction. Non-induced hASCs were used as a blank group. **f** Oil Red O staining and quantification of positively stained areas among primary I-hASCs and E-hASCs after adipogenic induction. **g** Relative expression of osteogenic genes in primary I-hASCs and E-hASCs after three weeks of osteogenic induction. Non-induced hASCs were used as a blank group. **h** Alizarin Red S staining and quantification of positively stained areas among primary I-hASCs and E-hASCs after three weeks of osteogenic induction. **i** Schematic of treatment with fourth-generation hASCs in full-thickness skin defects in mice. **j** Representative wound images and quantification of wound healing rates in PBS-, I-hASC-, and E-hASC-treated wounds at the indicated time points. PBS-treated wounds were used as a blank group. **k** Representative photomicrographs and quantitative analyses of blood perfusion in the blank, I-hASC-treated, and E-hASC-treated groups at different time points. Normal skin before skin defects were created was defined as the “Normal” group. **l** Representative images of immunohistochemical (IHC) staining for MPO and quantification of MPO-positive cells in PBS-, I-hASC- and E-hASC-treated wound sections at three days postsurgery. **m** Representative images of Masson’s trichrome staining in PBS-, I-hASC- and E-hASC-treated wound sections and quantification of Masson-positive areas at 14 days post-surgery. **n** Schematic of treatment with fourth-generation hASCs in fat-grafted nude mice. **o** Representative images of surviving fat grafts and comparisons of the weights of surviving fat grafts two months after grafting. Fat grafting with PBS was used as a blank group. **p** Representative images of H&E-stained adipose tissue sections two months after fat grafting with PBS, I-hASCs, or E-hASCs. **q** Histological evaluation of the integrity, inflammation, fibrosis, cysts, and vacuoles of the fat grafts two months after grafting by H&E staining. *P < 0.05; **P < 0.01; and ***P < 0.001.

In this study, the functional characteristics of hASCs were systematically evaluated to determine the effects of aging on their biological properties. Serial passaging revealed significant differences in self-renewal capacity between infant-derived hASCs (I-hASCs) and elderly-derived hASCs (E-hASCs). I-hASCs exhibited significantly greater proliferative activity and reduced senescence at matched passages, demonstrating age-dependent attrition of stem cell renewal potential (Fig. 1a–c). Furthermore, primary I-hASCs and E-hASCs were analyzed to ensure consistency when comparing their biological attributes. Surface marker analysis of hASCs confirmed typical mesenchymal phenotypes (CD29^+^/CD44^+^/CD90^+^/CD105^+^/CD34^−^), with I-hASCs exhibiting greater proportions of CD90^+^ (99.8% vs 87.6%) and CD105^+^ (98.6% vs 90.5%) cells (Supplementary Fig. S1a). Additionally, compared with E-hASCs, primary I-hASCs displayed superior cell activity, including faster migration, increased viability, enhanced morphology, and reduced apoptosis (Fig. 1d; Supplementary Fig. S1b, c). These results highlight the cellular activity and stemness advantages of primary I-hASCs over E-hASCs, suggesting that donor age significantly influences hASC functionality.

Adipogenic and osteogenic differentiation assays were performed to further evaluate the lineage plasticity of hASCs. After adipogenic induction, compared with the primary E-hASCs, the primary I-hASCs presented significantly higher expression of adipogenesis-related genes, including proliferator-activated receptor γ (PPARγ), adipocyte protein 2 (AP2), and CCAAT/enhancer-binding protein alpha (C/EBPα) (Fig. 1e). This transcriptional upregulation was corroborated by the results of adipogenic induction assays, which revealed increased lipid droplet formation in I-hASCs (Fig. 1f). In contrast, E-hASCs displayed reduced adipogenic potential, as evidenced by lower expression levels of related genes and fewer lipid droplets. Primary I-hASCs also demonstrated superior osteogenic differentiation capacity, as evidenced by the elevated expression of alkaline phosphatase (ALP) and Runt-related transcription factor 2 (RUNX2) and improved mineralization capacity (Fig. 1g, h). These findings indicate that donor age significantly impacts the multilineage differentiation potentials of hASCs, with younger donor-derived cells exhibiting enhanced adipogenic and osteogenic differentiation capabilities.

### The potential of elderly-derived hASCs to enhance wound healing and fat graft survival is diminished

The potential of I-hASCs and E-hASCs to improve wound healing and retain fat after transplantation was investigated in vivo (Fig. 1i and n). Before evaluating the effects of hASCs on wound healing and fat retention, we investigated the in vivo distribution, migration, and survival duration of mCherry-labeled I-hASCs and E-hASCs in different animal models (Supplementary Fig. S2a). Following the injection of mCherry-labeled hASCs at the wound boundary, these cells migrated non-uniformly from the periphery to the center (Supplementary Fig. S2b). During the first three days, the average fluorescence intensities at the boundary and wound sites decreased nearly by half each day, with only trace fluorescence signals detectable by the third day (Supplementary Fig. S2d). Moreover, mCherry-labeled hASCs were relatively uniformly distributed within the transplanted subcutaneous adipose tissue (Supplementary Fig. S2c). The fluorescence intensity decreased rapidly during the first three weeks, with only weak fluorescence signals remaining after six weeks, while the fluorescence signals essentially disappeared at the initial detection threshold by the eighth week (Supplementary Fig. S2e). On these bases, the treatment efficiency of the I-hASCs and E-hASCs in terms of wound healing and fat graft survival were systematically evaluated.

Compared with wounds treated with fourth-generation E-hASCs and phosphate-buffered saline (PBS) (blank group), the wounds treated with fourth-generation I-hASCs healed faster and exhibited better blood flow perfusion (*P* value < 0.05; Fig. 1j, k). Myeloperoxidase (MPO) staining to evaluate neutrophil infiltration in the dermis indicated that the I-hASC and E-hASC treatments decreased MPO^+^ cell infiltration in the wounds, whereas the I-hASC treatment more effectively alleviated neutrophil infiltration and wound inflammation (Fig. 1l). Fourteen days after full-thickness skin excision, Masson’s trichrome staining indicated increased collagen deposition in wounds treated with I-hASCs, with an average Masson-positive area of 65.32%, compared with 50.19% for E-hASCs and 40.22% for PBS (Fig. 1m).

Two months after the grafts were successfully placed in nude mice, the weights of the fat grafts co-injected with fourth-generation I-hASCs (0.257 ± 0.019 g) were significantly greater than those co-injected with PBS (0.204 ± 0.017 g) or fourth-generation E-hASCs (0.170 ± 0.040 g) (Fig. 1o). Hematoxylin and eosin (H&E) staining revealed that compared with the other groups, the fat graft containing fat and I-hASCs exhibited superior survival, characterized by improved morphological integrity, uniform lipid droplets, and reduced inflammatory cell infiltration, fibrosis, and cyst/vacuole formation (*P* value < 0.05; Fig. 1p, q).

### Proteomics analysis of aging-associated features of hASCs

**Fig. 2:**
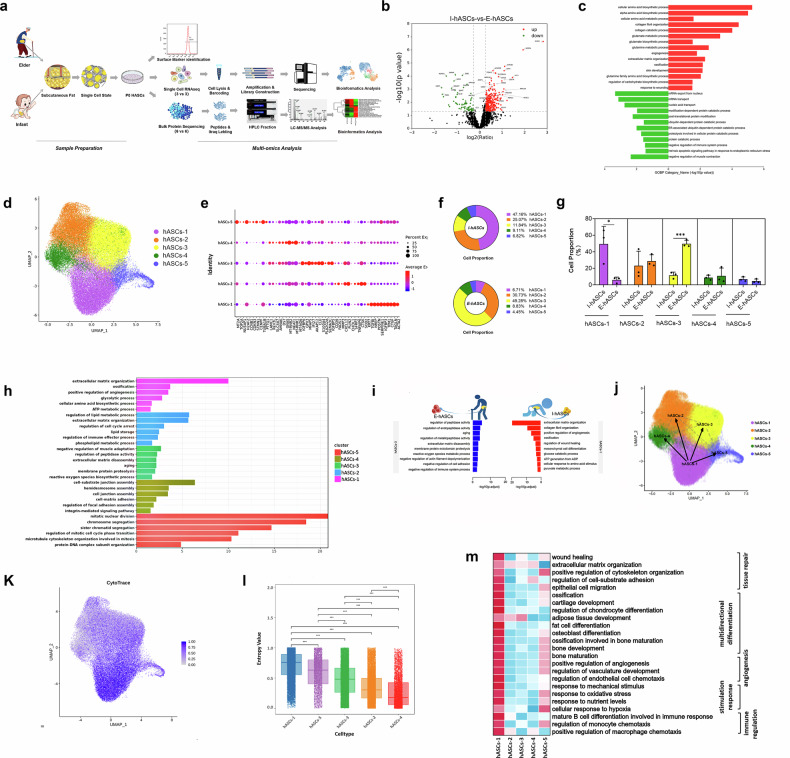
Proteomics and scRNA-seq revealed differences between I-hASCs and E-hASCs. **a** Workflow of primary hASC isolation, expansion, identification, and sequencing. **b** The DEPs between primary I-hASCs and E-hASCs displayed in a volcano plot. **c** The GO-BP pathways enriched in the DEPs for primary I-hASCs (red column) and E-hASCs (green column). **d** Five clusters of hASCs were visualized using UMAP. **e** Bubble plot presenting the top 10 hypervariable genes in each cell cluster of primary hASCs. **f** Ring plots presenting the proportions of the five cell clusters among primary I-hASCs and E-hASCs. **g** Differences in the proportion of cells in each cell cluster between primary I-hASCs and E-hASCs. **h** GO-BP pathways enriched in the hypervariable genes in each cluster of primary hASCs. **i** GO-BP pathways enriched in the upregulated DEGs in Cluster 1 I-hASCs (ACTA2^+^TAGLN^+^) and in Cluster 3 E-ASCs (IGF2BP5^+^SEMA3C^+^) are shown in the red and blue columns, respectively. **j** PAGA velocity graph of hASCs. **k** Feature plot based on the CytoTRACE scores. The scores range from 0 to 1, with higher scores indicating greater stemness. **l** CytoTRACE scores of 5 hASC subpopulations arranged in descending order and quantification of the CytoTRACE scores across the subpopulations. **m** Heatmap of UCell scores for evaluating pathway enrichment in the 5 cell subpopulations. ***P < 0.001.

The workflow for the isolation, surface marker identification, and sequencing of primary hASCs is illustrated in Fig. 2a. hASCs obtained from six infants and six older adults were subjected to protein sequencing using iTRAQ labeling. The mass error and peptide length distributions were used for quality control of each identified peptide (Supplementary Fig. S3a, b). Principal component analysis (PCA) and sample repeatability analysis revealed differential expression patterns between I-hASCs and E-hASCs from different donors (Supplementary Fig. S3c, d). When the I-hASCs were compared to the E-hASCs, 343 proteins were upregulated, whereas 69 were downregulated (Fig. 2b). Gene Ontology biological process (GO-BP) enrichment analysis of the differentially expressed proteins (DEPs) revealed their significant enrichment in pathways associated with hASC activity, ossification, wound healing, and angiogenesis (Fig. 2c). In addition, amino acid biosynthesis and metabolism were significantly enriched in the DEPs in I-hASCs, particularly in pathways related to glutamate and glutamine metabolism (Fig. 2c). These findings suggest a potential role for amino acid metabolism, especially relating to glutamate/glutamine dynamics, in regulating the biological functions of hASCs.

### ScRNA-seq revealed the heterogeneity of hASCs and aging-associated features

scRNA-seq was performed to investigate the heterogeneity and aging-associated features of primary hASCs. hASCs were isolated from three infant donors (I-hASCs) and three elderly donors (E-hASCs), and their transcriptomic profiles were compared (Fig. 2a). Following stringent quality control, metrics including the number of detected genes per cell (nFeature RNA), total UMI count (nCount RNA), and mitochondrial read proportion (percent.mt) were determined to ensure data reliability (Supplementary Fig. S4a–c). Overall, 58,215 high-quality hASCs were retained downstream analysis, comprising 35,761 I-hASCs and 22,454 E-hASCs, with median gene counts ranging from 1812 to 2705 and median UMI counts ranging from 4,798 to 5,903. PCA revealed distinct clustering patterns between I-hASCs and E-hASCs that reflected age-associated differences (Supplementary Fig. S4d). Uniform manifold approximation and projection (UMAP) analysis revealed five distinct clusters within the hASC population, highlighting cellular heterogeneity (Fig. 2d). The top 10 highly variable genes (HVGs), identified by rank product analysis, were proposed as potential markers for each cluster, highlighting the unique transcriptional signatures of hASCs (Fig. 2e). The distribution of these clusters differed significantly between the I-hASCs and E-hASCs. Specifically, Cluster 1, characterized by elevated expression of actin alpha 2 (ACTA2) and transgelin (TAGLN), was predominantly found in I-hASCs (47.16%), whereas Cluster 3, characterized by high levels of insulin-like growth factor-binding protein 5 (IGFBP5) and semaphorin 3C (SEMA3C), was more prevalent in E-hASCs (49.28%) (*P* value < 0.05; Fig. 2f, g).

GO-BP enrichment analyses of the HVGs in each cluster provided insights into their functional roles (Fig. 2h). Cluster 1 (ACTA2^+^TAGLN^+^) was primarily associated with pathways related to ossification, angiogenesis, and various metabolic processes. Cluster 2 was enriched in lipid metabolic pathways. Cluster 3 exhibited senescence-associated pathways, including aging, protein proteolysis, and extracellular matrix disassembly. Cluster 4 was involved in cell junction and adhesion pathways, whereas Cluster 5 was primarily associated with cell mitotic division and the cell cycle.

Supplementary Fig. S4e depicts the differences in enriched GO-BP pathways between I-hASCs and E-hASCs across the five hASC clusters, revealing the commonalities among the enriched pathways in different cell clusters of primary I-hASCs or E-hASCs. ATP metabolic processes were enriched in all the clusters of I-hASCs, and collagen fibril organization, mRNA catabolic processes, NADH and glucose metabolic processes, and the regulation of wound healing were enriched in multiple clusters. In E-hASCs, the aging pathway was enriched in all clusters, and pathways related to extracellular matrix disassembly and peptidase regulation were detected in various clusters. Furthermore, we highlight the enriched pathways of Cluster 1 in I-hASCs and Cluster 3 in E-hASCs (Fig. 2i), which are consistent with the enriched pathways observed in Clusters 1 and 3 (Fig. 2h). Gene set enrichment analysis (GSEA) revealed significant enrichment of the biosynthesis of amino acids, glycolysis/gluconeogenesis, and oxidative phosphorylation in hASC Cluster 1, with a normalized enrichment score > 1 and *P* value ≤ 0.05 (Supplementary Fig. S4f). Overall, a decrease in the proportion of cells in Cell Cluster 1 and an increase in the proportion of cells in Cluster 3 may be closely associated with age-related changes in hASCs.

### Functional characteristics of the ACTA2^+^TAGLN^+^ subset of hASCs

Given that the ACTA2^+^TAGLN^+^ subset of hASCs is the dominant I-hASC subpopulation, we conducted a systematic analysis of the developmental potential, stemness, and functional plasticity of this subset. Pseudotime analysis through the partition-based graph abstraction (PAGA) method and RNA velocity analysis revealed that the ACTA2^+^TAGLN^+^ subset served as the initial point of the developmental trajectory in hASCs, leading to the formation of the other subpopulations (Fig. 2j; Supplementary Fig. S4g). Additionally, the latent time feature plot confirmed the early emergence of the ACTA2^+^TAGLN^+^ subset among the hASCs (Supplementary Fig. S4h). Moreover, CytoTRACE score evaluation revealed increased stemness in the ACTA2^+^TAGLN^+^ subset compared with that of other subpopulations, suggesting its significance as the developmental origin of hASCs (Fig. 2k, l). Furthermore, the UCell method was used to explore the functional plasticity of the ACTA2^+^TAGLN^+^ subset. A heatmap analysis revealed that the tissue repair, multidirectional differentiation, angiogenesis, stimulation response, and immune regulation scores were superior in the ACTA2^+^TAGLN^+^ subset compared with those in the other subpopulations (Fig. 2m). Overall, the ACTA2^+^TAGLN^+^ subset, which serves as the developmental starting point of hASCs, exhibited significantly better developmental potential, stemness, and functional plasticity.

### Cell–cell interaction and transcription factor (TF) regulation in the five clusters of I-hASCs and E-hASCs

Cell–cell interactions among the five clusters in primary I-hASCs and E-hASCs revealed distinct characteristics on the basis of ligand–receptor pairs. In the I-hASCs, Cluster 1 exhibited close interactions with Clusters 2 and 3, with 63 and 65 ligand–receptor pairs, respectively (Supplementary Fig. S4i). Conversely, in E-hASCs, a close interaction between Clusters 2 and 3 was observed, supported by 65 ligand–receptor pairs (Supplementary Fig. S4j). Supplementary Fig. S4k–l depict the top 30 ligand–receptor pairs between Cluster 1 and the other four cell clusters in I-hASCs and E-hASCs. Differences in factor expression and activity were identified in Clusters 1 and 3 I-hASCs and E-hASCs using the Python implementation of SCENIC (PySCENIC) (Supplementary Fig. S4m, n). Y-box binding protein 1 (YBX1) was highly expressed specifically in Cluster 1 of I-hASCs and was associated with high regulon activity (Supplementary Fig. S4m). YBX1 usually interacts with insulin-like growth factor II mRNA binding proteins (IGF2BPs) to stabilize m6A-modified transcripts, thereby coordinating cell proliferation, survival, and chromatin destabilization, highlighting the potential of the YBX1-IGF2BP axis in regulating I-hASC stemness and activity^30^.

### Differences in age-related metabolic patterns between I-hASCs and E-hASCs

**Fig. 3:**
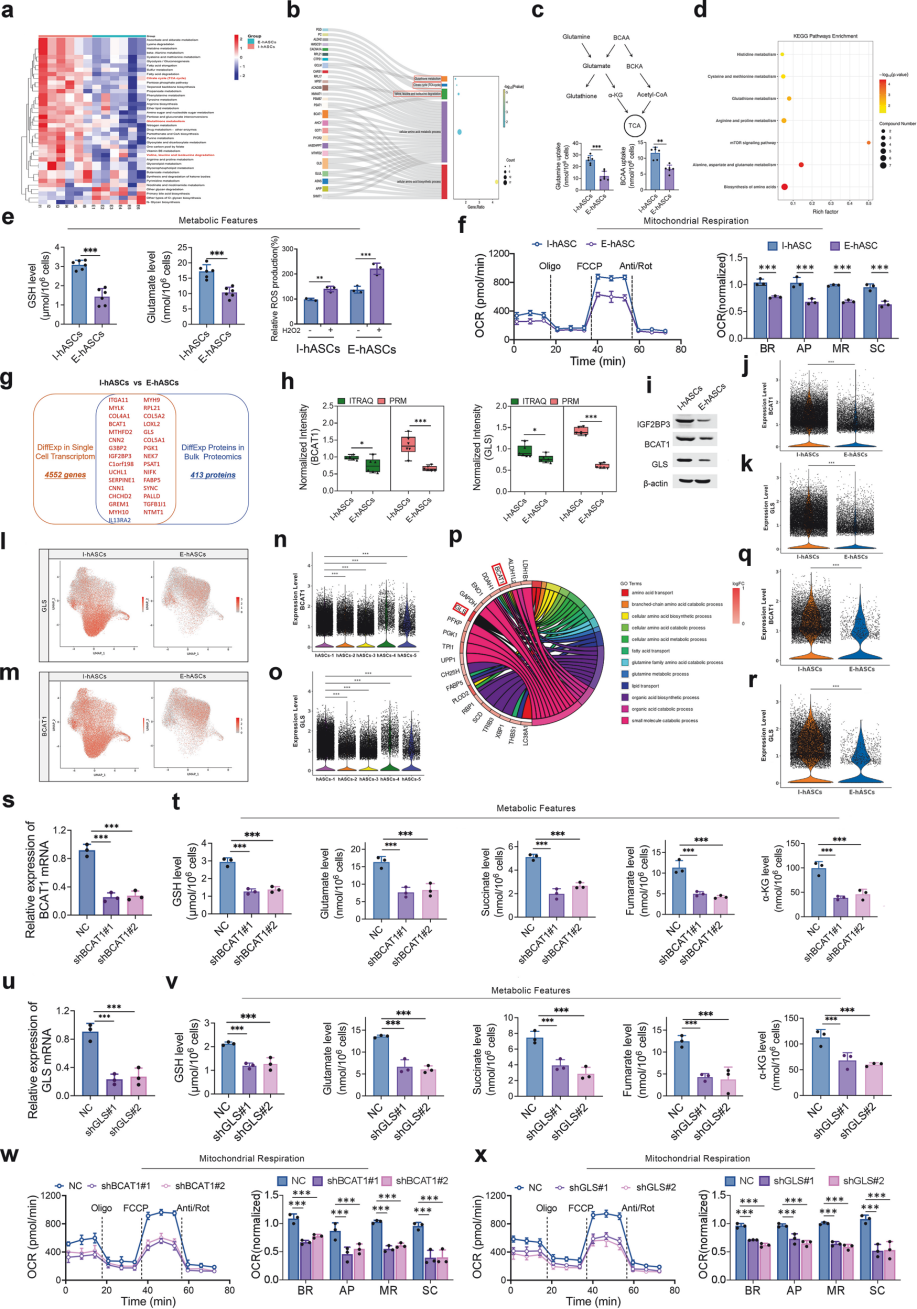
The downregulation of BCAT1 and GLS compromised glutamine and BCAA metabolism in E-hASCs. **a** Heatmap presenting the KEGG metabolic pathways enriched in each sample based on the proteomics data. **b** Sankey bubble diagram showing the enrichment status of the specific metabolic pathways in primary I-hASCs and the DEPs involved. **c** Flowchart of glutamine and BCAA metabolism in and glutamine and BCAA uptake capacities of primary I-hASCs and E-hASCs. **d** KEGG enrichment analysis of aging-related metabolic pathways between primary I-hASCs and E-hASCs according to the metabolomic data. **e** Differences in GSH and glutamate levels and H_2_O_2_-induced ROS production between primary I-hASCs and E-hASCs. **f** A high-throughput Seahorse assay was used to monitor the cellular OCR and quantify the basal OCR (BR), ATP-linked OCR (AP), maximal OCR (MR), and spare OCR (SC) in primary I-hASCs and E-hASCs. **g** Venn diagram displaying the intersection of 4,552 DEGs and 413 DEPs between I-hASCs and E-hASCs at the bulk level. DEGs and DEPs are indicated in red and blue, denoting common upregulation and downregulation, respectively. **h** Differences in BCAT1 and GLS protein expression between primary I-hASCs and E-hASCs detected using iTRAQ and PRM. **i** IGF2BP3, BCAT1, and GLS protein expression in primary I-hASCs and E-hASCs detected using western blotting. **j, k** Differential expression of BCAT1 (**j**) and GLS (**k**) mRNAs between primary I-hASCs and E-hASCs determined using scRNA-seq. **l, m** Feature plots of BCAT1 (**l**) and GLS (**m**) mRNA expression in I-hASCs and E-hASCs based on the scRNA-seq data. BCAT1 (**n**) and GLS (**o**) mRNA expression among the five hASC clusters. **p** Chord diagram displaying the enriched metabolic pathways and upregulated DEGs in Cluster 1 I-hASCs. Differential expression of BCAT1 (**q**) and GLS (**r**) mRNAs between Cluster 1 I-hASCs and E-hASCs. **s** Validation of BCAT1 knockdown using qRT–PCR after transfection of two distinct short hairpin RNA lentiviruses targeting BCAT1 (shBCAT1#1 and shBCAT1#2) into I-hASCs. **t** GSH, glutamate, succinate, fumarate, and α-KG levels in I-hASCs with or without BCAT1 knockdown. **u** Validation of GLS knockdown using qRT–PCR after I-hASCs were transfected with two distinct short hairpin RNA lentiviruses targeting GLS (shGLS#1 and shGLS#2). **v** GSH, glutamate, succinate, fumarate, and α-KG levels in I-hASCs with or without GLS knockdown. OCRs of I-hASCs with and without BCAT1 (**w**) or GLS (**x**) knockdown and quantification of the BR, AP, MR, and SC in each group. **P < 0.01; ***P < 0.001.

Proteomic profiling of primary hASCs revealed age-dependent differences in the levels of BCAAs and GSH and the activities of glycolysis/gluconeogenesis and the TCA cycle, with I-hASCs exhibiting upregulation of key enzymes related to BCAAs (BCAT1) and glutamine-related metabolic pathways (GLS) compared with their levels in E-hASCs (Fig. 3a, b). Glutamine and BCAAs serve as primary donors for GSH synthesis and the TCA cycle by generating glutamate and branched chain α-ketoacids (BCKAs), thereby maintaining redox homeostasis and energy production, as corroborated by elevated substrate uptake by I-hASCs (glutamine: 2.16-fold; BCAA: 1.76-fold, *P* value < 0.01; Fig. 3c). Analysis of metabolic pathways further demonstrated the selective enrichment of factors related to amino acid biosynthesis, GSH, and glutamate metabolism in I-hASCs (Fig. 3d). Targeted quantification analysis revealed significant age-dependent reductions in the levels of glutamate (1.67-fold, *P* value < 0.001) and GSH (2.15-fold, *P* value < 0.001) in E-hASCs, which was associated with impaired ROS clearance (Fig. 3e). Furthermore, Seahorse assays revealed elevated mitochondrial respiration in primary I-hASCs, as indicated by an increased cellular oxygen consumption rate (OCR), as determined by increased basal respiration, ATP production, maximal respiration, and spare capacity (Fig. 3f). These data establish that attenuated glutamine and BCAA metabolism underlies redox imbalance and bioenergetic collapse in aged hASCs.

### The distinctive expression patterns of BCAT1 and GLS contributed to age-related metabolic reprogramming in hASCs

Here, we established the significance of impaired glutamine and BCAA metabolism during hASC aging. Furthermore, we identified 31 genes whose expression significantly differed between primary I-hASCs and E-hASCs through integrated scRNA-seq and bulk proteomics analyses (Fig. 3g). We detected significant upregulation of BCAT1 and GLS at both the mRNA and protein levels in I-hASCs compared with their levels in E-hASCs. Such upregulation was validated at the mRNA level using scRNA-seq and reverse transcriptase quantitative polymerase chain reaction (RT–qPCR) and at the protein level using parallel reaction monitoring (PRM) and western blotting (Fig. 3h–k; Supplementary Fig. S5a, b). The feature plots revealed the predominant expression of BCAT1 and GLS mRNA in I-hASC Cluster 1 (Fig. 3l–o). Additionally, chord diagram analysis revealed significant increases in BCAT1 and GLS expression across 12 GO terms associated with amino acid metabolic processes, particularly in BCAA and glutamine metabolism, within Cluster 1 (Fig. 3p). BCAT1 and GLS expression levels were also significantly elevated in I-hASC Cluster 1 compared with those in E-hASC Cluster 1 (Fig. 3q, r).

The metabolites associated with BCAA and glutamine metabolism in hASCs were assessed following BCAT1 and GLS knockdown to comprehensively elucidate the pivotal roles of BCAT1 and GLS in regulating hASC metabolism. Glutamate and GSH levels decreased significantly after BCAT1 or GLS knockdown, underscoring the involvement of BCAA and glutamine catabolism in the redox homeostasis of hASCs facilitated by BCAT1 and GLS (Fig. 3s–v). In addition, reduced succinate, fumarate, and α-ketoglutarate (α-KG) levels indicated the contribution of BCAT1 and GLS to the TCA cycle and energy production in hASCs via BCAA and glutamine catabolism (Fig. 3t, v). Knocking down BCAT1 or GLS expression in hASCs significantly reduced the OCR, basal respiration, ATP production, maximal respiration, and spare capacity of the mitochondria (Fig. 3w, x). As a result, the downregulation of BCAT1 or GLS in E-hASCs could precipitate redox homeostasis and energy production decline, ultimately influencing hASC activity.

### BCAT1 and GLS regulation of age-related hASC activity is mediated by IGF2BP3

**Fig. 4:**
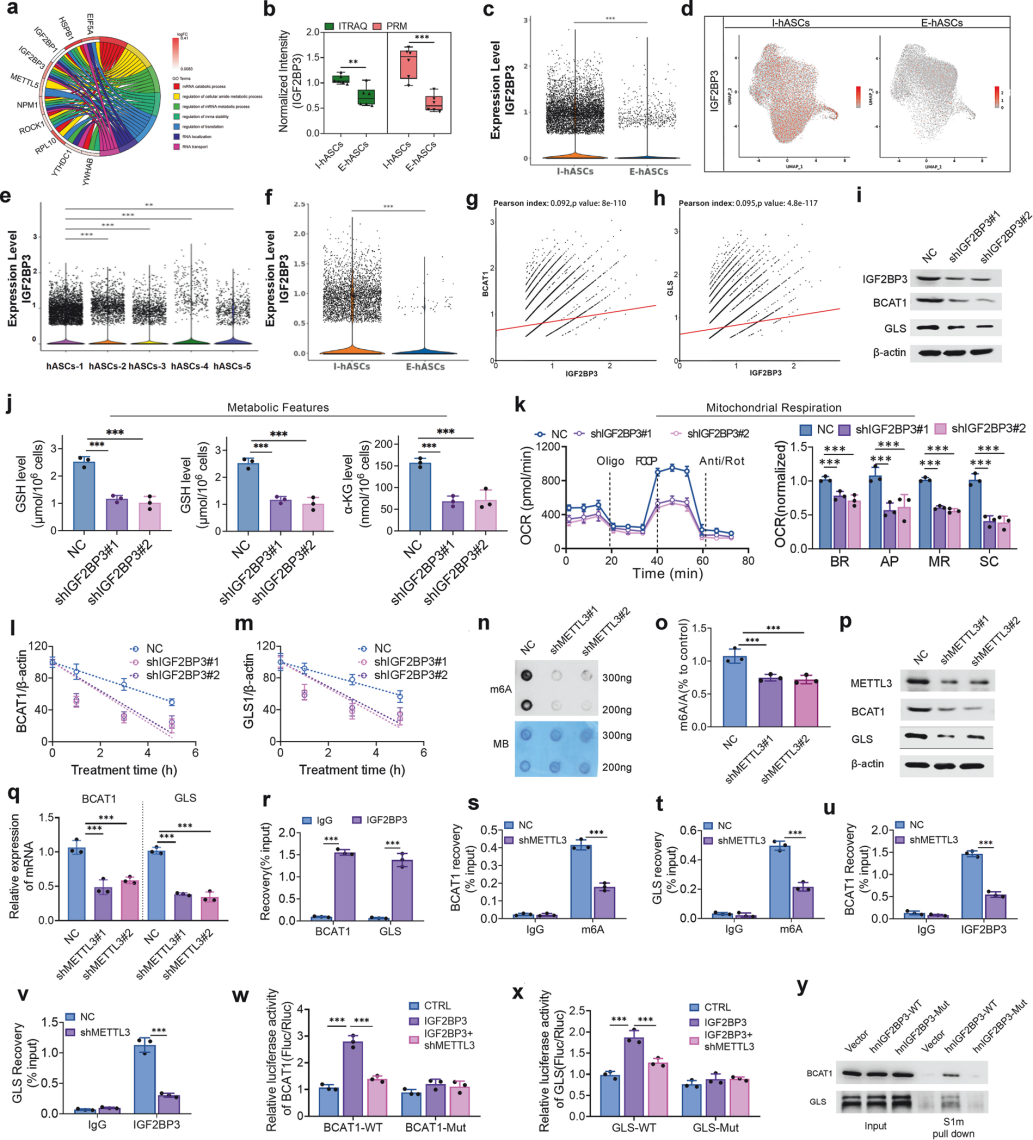
IGF2BP3 stabilizes BCAT1/GLS mRNAs in hASCs via METTL3-mediated m6A modification. **a** Chord diagram showing that the highly expressed IGF2BP3 protein in I-hASCs is primarily involved in mRNA metabolism and stability pathways. **b** Differences in IGF2BP3 protein expression between I-hASCs and E-hASCs detected using iTRAQ and PRM. **c** Differential expression of IGF2BP3 mRNA between I-hASCs and E-hASCs using scRNA-seq. **d** Feature plots of IGF2BP3 mRNA expression in I-hASCs and E-hASCs based on the scRNA-seq data. **e** IGF2BP3 mRNA expression among the five hASC clusters, including I-ASCs and E-hASCs. **f** Deferential expression of IGF2BP3 mRNA between I-hASCs and E-hASCs in Cluster 1. **g**, **h** Pearson correlations of IGF2BP3 and BCAT1 mRNA expression (**g**) and of IGF2BP3 and GLS mRNA expression (**h**) in the total hASCs. **i** Western blotting assay was used to detect the protein expression of IGF2BP3, BCAT1, and GLS in I-hASCs after they were transfected with two distinct short hairpin RNA lentiviruses targeting IGF2BP3 (shIGF2BP3#1 and shIGF2BP3#2). **j** GSH, glutamate, and α-KG levels in I-hASCs with or without IGF2BP3 knockdown. **k** OCRs of I-hASCs with and without IGF2BP3 knockdown and quantification of the BR, AP, MR, and SC in each group. **l**, **m** Stability of BCAT1 (**l**) and GLS (**m**) mRNA in I-hASCs with or without IGF2BP3 knockdown. IGF2BP3 transcription was inhibited with actinomycin. **n** The m6A dot-blot assay revealed global RNA m6A levels after I-hASCs were transfected with two distinct short hairpin RNA lentiviruses targeting METTL3 (shMETTL3#1 and shMETTL3#2). Methylene blue staining served as a loading control. **o** m6A levels in I-hASCs with or without METTL3 knockdown. **p** Western blotting was used to detect METTL3, BCAT1, and GLS protein expression in I-hASCs after METTL3 knockdown. **q** qRT–PCR was used to measure BCAT1 (left panel) and GLS (right panel) mRNA expression levels in I-hASCs with or without METTL3 knockdown. **r** RIP assays in I-hASCs were used to assess the specific binding of IGF2BP3 to m6A-modified sites on BCAT1 mRNA (+1221 locus) and GLS mRNA (+4376 locus), and an anti-IGF2BP3-specific antibody was used for immunoprecipitation; an IgG antibody was used as a negative control. Analysis of MeRIP assays in I-hASCs to detect the recovery of BCAT1 mRNA +1221 locus (**s**) and GLS mRNA +4376 locus (**t**) with an anti-m6A antibody after METTL3 knockdown. **u**, **v** RIP assays were performed using an anti-IGF2BP3 antibody or IgG in I-hASCs after METTL3 knockdown. Luciferase activities of WT or Mut BCAT1 (**w**) and GLS (**x**) reporter vectors were quantified in I-hASCs under defined genetic perturbations: control, IGF2BP3 overexpression (IGF2BP3), or IGF2BP3 overexpression with METTL3 knockdown (IGF2BP3+shMETTL3). **y** IGF2BP3 was performed using streptavidin beads after S1m-WT/Mut-tagged BCAT1 and GLS mRNA were expressed. **P < 0.01; ***P < 0.001.

We explored the underlying mechanism of reduced BCAT1 and GLS levels in aged hASCs. GO-BP enrichment analysis of the proteomic data using chord diagrams revealed that IGF2BP3 plays key roles in pathways related to mRNA stability, metabolic processes, and translation (Fig. 4a). Moreover, an integrative analysis of the transcriptomic and proteomic data revealed the upregulation of IGF2BP3 in primary I-hASCs (Fig. 3g). The elevated IGF2BP3 protein expression in I-hASCs was confirmed using PRM and western blotting (Figs. 3i and 4b). The upregulation of IGF2BP3 expression was also proven at the mRNA level using scRNA-seq and RT–qPCR (Fig. 4c; Supplementary Fig. S5c). Like those for BCAT1 and GLS, the feature plots and bar plots revealed that IGF2BP3 mRNA was expressed mainly in I-hASC Cluster 1 (Fig. 4d–f). Moreover, correlation analysis revealed a significant positive correlation between the mRNA expression of BCAT1 or GLS and that of IGF2BP3 in each cell, with a *P* value < 0.05 (Fig. 4g, h). Moreover, in vitro enzyme activity assays confirmed that IGF2BP3 does not notably affect the enzymatic functions of BCAT1 or GLS (Supplementary Fig. S5d, e). These findings suggest that IGF2BP3-mediated RNA metabolism regulates BCAT1 and GLS expression, influencing hASC activity.

We employed two distinct short hairpin RNA lentiviruses targeting IGF2BP3 to knock down its expression in I-hASCs and thoroughly investigate the roles of IGF2BP3 in glutamine and BCAA metabolism during hASC aging. Western blotting confirmed a coordinated decrease in GLS and BCAT1 levels following IGF2BP3 knockdown (Fig. 4i). Furthermore, glutamine and BCAA catabolism were impeded upon IGF2BP3 knockdown in hASCs, as evidenced by the levels of GSH, glutamate, succinate, fumarate, and α-KG and ATP production (Fig. 4j; Supplementary Fig. S5f–h). Additionally, IGF2BP3 knockdown in hASCs suppressed mitochondrial respiration, as indicated by reductions in the OCR, including basal respiration, ATP production, maximal respiration, and spare capacity (Fig. 4k). Moreover, IGF2BP3 knockdown facilitated the degradation of BCAT1 and GLS mRNAs, as demonstrated by the measured mRNA half-lives of these genes following transcriptional inhibition with actinomycin D (Fig. 4l, m). These findings indicate that IGF2BP3 may play a crucial role in triggering glutamine and BCAA metabolism in hASCs by stabilizing GLS and BCAT1 mRNAs.

To validate the roles of IGF2BP3 in the m6A modification of BCAT1 and GLS mRNAs in hASCs more rigorously, IGF2BP3 knockout (KO) was induced in immortalized hASCs (ADHX-C1061). Similar results were observed with IGF2BP3-KO hASCs, which exhibited significant decreases in the mRNA and protein levels of BCAT1 and GLS (Supplementary Fig. S6a–c). Metabolite analysis revealed a notable reduction in the GSH, glutamate, and α-KG levels in the IGF2BP3-KO hASCs (Supplementary Fig. S6d–f). Additionally, IGF2BP3 depletion in hASCs resulted in a marked decrease in the OCR, indicating impaired mitochondrial respiratory function (Supplementary Fig. S6g). Moreover, the mRNA stability of BCAT1 and GLS was significantly reduced in IGF2BP3-KO hASCs, which is consistent with previous findings demonstrating the role of IGF2BP3 in stabilizing these mRNAs (Supplementary Fig. S6h, i).

### IGF2BP3 stabilizes BCAT1 and GLS transcripts through METTL3-mediated m6A modification

IGF2BP3 acts as a mediator of m6A-dependent nuclear RNA processing^31^. We hypothesized that IGF2BP3 modulates the stability of BCAT1 and GLS mRNAs in an m6A-dependent manner. To validate this hypothesis, we suppressed the expression of methyltransferase 3, N6-adenosine-methyltransferase (METTL3) in I-hASCs using two distinct short hairpin RNA lentiviruses. METTL3 knockdown reduced overall m6A levels in hASCs, indicating the successful lentivirus-mediated suppression of m6A modification (Fig. 4n, o). Moreover, METTL3 knockdown reduced BCAT1 and GLS expression at the protein and mRNA levels (Fig. 4p, q).

We predicted the top five m6A sites on BCAT1 and GLS mRNAs using the sequence-based RNA adenosine methylation site predictor (SRAMP) online tool (http://www.cuilab.cn/sramp) (Supplementary Fig. S7a, b). These predictions were confirmed using methylated RNA immunoprecipitation (MeRIP) assays. The most enriched regions following m6A antibody pull-down were identified at position +1221 in the 3’ UTR of BCAT1 and at position +4376 in the 3’ UTR of GLS (Supplementary Fig. S7c, d), indicating that GLS and BCAT1 mRNAs undergo m6A modification, which contributes to their relative stability. RNA immunoprecipitation (RIP) assays revealed that the anti-IGF2BP3 antibody enriched BCAT1 and GLS mRNA (Fig. 4r), indicating an interaction between IGF2BP3 and BCAT1 mRNA and between IGF2BP3 and GLS mRNA in hASCs. MeRIP assays demonstrated that silencing METTL3 reduced m6A of BCAT1 and GLS mRNA (Fig. 4s, t). In addition, METTL3 knockdown contributed to reduced IGF2BP3 enrichment (Fig. 4u, v), suggesting that m6A modifications on BCAT1 and GLS mRNA are crucial for IGF2BP3 recognition. To validate these results, we constructed firefly luciferase reporters for GLS and BCAT1 with either wild-type (WT) or mutant (Mut) m6A site sequences (Supplementary Fig. S7e, f). IGF2BP3 expression significantly increased luciferase activity with the BCAT1-WT and GLS-WT reporters, but this effect was abolished by mutation of the m6A site (Fig. 4w, x). However, METTL3 knockdown blocked the IGF2BP3-mediated increase in luciferase activity (Fig. 4w, x). Additionally, we transfected hASCs with an S1m-tagged BCAT1 or GLS construct to allow m6A modification. Mutation of the m6A sites in BCAT1 and GLS disrupted their relative association with IGF2BP3, as determined by streptavidin aptamer capture (Fig. 4y).

### IGF2BP3 governs hASC stemness and regenerative capacity through METTL3-dependent m6A modification

**Fig. 5:**
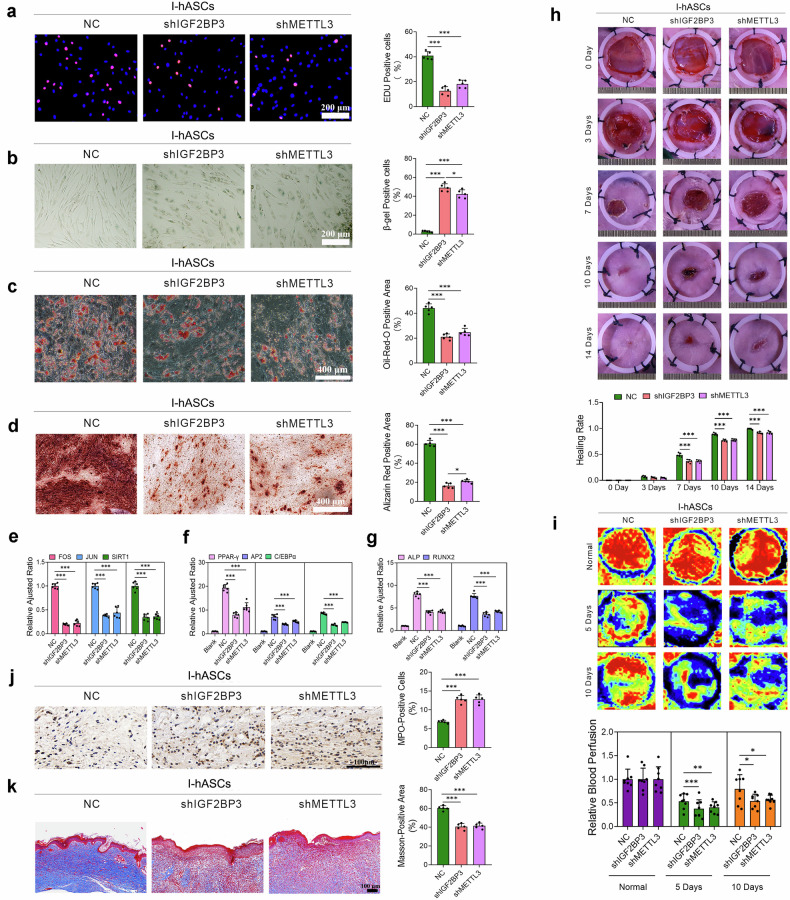
The downregulation of IGF2BP3 or METTL3 decreased the viability, lineage plasticity, and tissue remodeling potentials of I-hASCs. **a** Representative images of EdU staining and quantification of the percentage of EdU-positive I-hASCs transfected with shIGF2BP3#1 or shMETTL3#1. **b** SA-β-gal staining and quantification of the percentages of SA-β-gal-positive I-hASCs transfected with shIGF2BP3#1 or shMETTL3#1. **c** Oil Red O staining and quantification of the positively stained areas of I-hASCs transfected with shIGF2BP3#1 or shMETTL3#1 after three weeks of adipogenic induction. **d** Alizarin Red S staining and quantification of the positively stained areas of I-hASCs transfected with shIGF2BP3#1 or shMETTL3#1 after three weeks of osteogenic induction. **e** Relative expression of age-related genes in I-hASCs transfected with shIGF2BP3#1 or shMETTL3#1. **f** Relative expression of lipogenesis-related genes in I-hASCs transfected with shIGF2BP3#1 or shMETTL3#1 after adipogenic induction. **g** Relative expression of osteogenesis-related genes in I-hASCs transfected with shIGF2BP3#1 or shMETTL3#1 after osteogenic induction. **h** Representative wound images and quantification of wound healing rates are at different time points after injection of I-hASCs transfected with shIGF2BP3#1 or shMETTL3#1. **i** Representative photomicrographs and quantitative analyses of wound blood perfusion at different time points after injection of I-hASCs transfected with shIGF2BP3#1 or shMETTL3#1. **j** Representative images of MPO-positive cells determined by IHC staining and quantification of MPO-positive cells in wound sections after injection of I-hASCs transfected with shIGF2BP3#1 or shMETTL3#1 three days post-surgery. **k** Representative images of Masson’s trichrome-stained wound sections after injection of I-hASCs transfected with shIGF2BP3#1 or shMETTL3#1, and Masson-positive areas were quantified at 14 days post-surgery. *P < 0.05; **P < 0.01; and ***P < 0.001.

Previous studies have indicated that alterations in various metabolic pathways, such as GSH metabolism, are pivotal in driving bimodality during stem cell aging^15^. Given that IGF2BP3 can modulate glutamine and BCAA metabolism by stabilizing BCAT1 and GLS mRNAs (Fig. 4i–y), its impact on the senescence and stemness of hASCs was investigated. EdU assays and calcein-AM/PI staining revealed that IGF2BP3 knockdown significantly reduced the proliferative ability and altered the morphology of hASCs (Fig. 5a; Supplementary Fig. S8a). After IGF2BP3 knockdown, hASC senescence and apoptosis were exacerbated, while the migration and adipogenic and osteogenic differentiation of these cells were impaired (Fig. 5b–g; Supplementary Fig. S8b, c). To validate the effects of IGF2BP3-deficient hASCs on wound healing, we subcutaneously injected hASCs into murine wound margins and monitored tissue regenerative outcomes. Compared with the control hASCs, wounds treated with IGF2BP3-deficient hASCs exhibited slower healing rates and reduced blood flow perfusion (Fig. 5h, i). Staining of wound sections revealed that compared with control hASC injection, injection of hASCs with IGF2BP3 knockdown increased the proportion of MPO^+^ cells in the early stages of wound healing and decreased collagen deposition (Fig. 5j, k).

Having established the critical role of IGF2BP3 in maintaining the characteristics of hASCs, we next performed METTL3 knockdown experiments to investigate the m6A-dependent regulation of hASC stemness, differentiation potential and regenerative capacity. Consistent with the above data, METTL3 knockdown decreased hASC proliferation, migration, and adipogenic and osteogenic differentiation capacity but increased senescence and apoptosis (Fig. 5a–g; Supplementary Fig. S8a–c). Analysis of hASCs post-METTL3 silencing in mice revealed that IGF2BP3 in hASCs facilitates wound healing in an m6A-dependent manner, possibly through METTL3 (Fig. 5h–k).

### BCAT1 and GLS mediate IGF2BP3-driven hASC rejuvenation to sustain their functionality

**Fig. 6:**
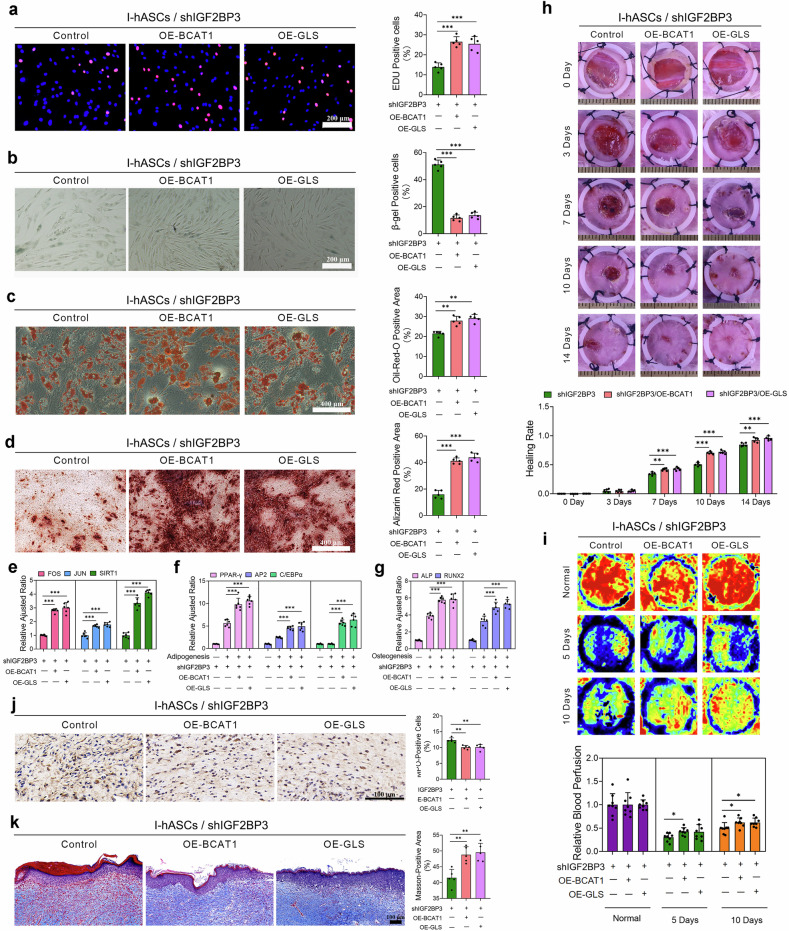
BCAT1 and GLS overexpression restored the viability, lineage plasticity, and tissue remodeling potentials of shIGF2BP3-transfected I-hASCs. **a** Representative images of EdU staining and quantification of the percentage of EdU-positive hASCs transfected with shIGF2BP3#1 with BCAT1 or GLS overexpression. **b** SA-β-gal staining and quantification of the percentage of SA-β-gal-positive hASCs transfected with shIGF2BP3#1 with BCAT1 or GLS overexpression. **c** Oil Red O staining and quantification of the positively stained areas of hASCs transfected with shIGF2BP3#1 with BCAT1 or GLS overexpression after adipogenic induction. **d** Alizarin Red S staining and quantification of positively stained areas of hASCs transfected with shIGF2BP3#1 with BCAT1 or GLS overexpression after osteogenic induction. **e** Relative expression of age-related genes in hASCs in each group. **f** Relative expression of lipogenesis-related genes in hASCs after adipogenic induction in each group. **g** Relative expression of osteogenesis-related genes in hASCs after osteogenic induction in each group. **h** Representative wound images and quantification of wound healing rates after the injection of shIGF2BP3#1-transfected hASCs (before and after BCAT1 or GLS overexpression) at different time points. **i** Representative photomicrographs and quantitative analyses of wound blood perfusion after the injection of shIGF2BP3#1-transfected hASCs (before and after BCAT1 or GLS overexpression) at different time points. **j** Representative images of MPO-positive cells by IHC staining and quantification of MPO-positive cells in wound sections after the injection of shIGF2BP3#1-transfected hASCs (before and after BCAT1 or GLS overexpression) three days post-surgery. **k** Representative images of Masson’s trichrome-stained wound sections after the injection of shIGF2BP3#1-transfected hASCs (before and after BCAT1 or GLS overexpression) and quantification of Masson-positive areas at 14 days post-surgery. *P < 0.05; **P < 0.01; and ***P < 0.001.

We performed genetic rescue experiments after IGF2BP3 knockdown to validate the necessity of BCAT1 and GLS in IGF2BP3-mediated hASC rejuvenation, followed by western blotting (Supplementary Fig. S9a). BCAT1 or GLS overexpression partially reversed the aging-associated phenotypes of IGF2BP3-silenced hASCs, including increased proliferation, migration, and adipogenic and osteogenic differentiation potential, enhanced morphology, and reduced senescence and apoptosis (Fig. 6a–g; Supplementary Fig. S9b–d). In vivo experiments further corroborated these findings: IGF2BP3-depleted hASCs with BCAT1 or GLS overexpression exhibited accelerated wound closure rates, enhanced tissue remodeling, reduced inflammatory infiltration, and increased collagen deposition (Fig. 6h–k). These data identify BCAT1 and GLS as downstream effectors through which IGF2BP3 sustains the stemness and regenerative capacity of hASCs, highlighting metabolic adaptation as a potential target for counteracting hASC aging.

### IGF2BP3 overexpression reverses age-associated dysfunction of E-hASCs

Rescue experiments were conducted to explore the potential of IGF2BP3 to maintain hASC metabolic homeostasis and activity and reverse age-associated dysfunction of E-hASCs. In E-hASCs, elevated IGF2BP3 levels significantly promoted the mRNA and protein expression of BCAT1 and GLS, which are critical enzymes in BCAA and glutamine metabolism, respectively, by increasing the stability of their mRNAs (Supplementary Fig. S10a–c, h, i). The catabolism of glutamine and BCAAs was subsequently restored in E-hASCs overexpressing IGF2BP3, with increased levels of GSH, glutamate, and α-KG, suggesting that metabolic homeostasis was restored (Supplementary Fig. S10d–f). Furthermore, IGF2BP3 overexpression in E-hASCs led to significant recovery of the OCR, indicating the resumption of mitochondrial respiratory function (Supplementary Fig. S10g). These in vitro findings collectively indicate that IGF2BP3 plays a pivotal role in restoring metabolic homeostasis in E-hASCs.

Additionally, IGF2BP3 overexpression partially reversed the age-related phenotypes of E-hASCs by increasing their proliferation, migration, and adipogenic and osteogenic differentiation capacity, improving their morphology, and upregulating adipogenic and osteogenic marker genes (Supplementary Fig. S11a, c–f, h, i). Such reversal was accompanied by reductions in senescence, the expression of senescence-associated genes, and the apoptosis rate (Supplementary Fig. S11b, g, j). Collectively, the results of the rescue experiments strongly suggested that IGF2BP3 plays a crucial role in restoring the functionality of E-hASCs, underscoring its significance as a key regulator of the activities, metabolic adaptations, and biological functions of hASCs.

### The regulation of up- and downstream of IGF2BP3 in hASCs during aging

DNA methylation has been well documented to be a key factor in human aging^32^. To further elucidate the underlying mechanism of age-dependent variations in IGF2BP3 expression, we collected whole blood samples from healthy individuals of different ages to capture overall aging features. We subsequently performed a comprehensive analysis of potential DNA methylation sites in the CpG island of the IGF2BP3 promoter on the basis of the Illumina 450 K DNA methylation microarray in a GEO dataset. A total of 228 samples were obtained in the Children group, 232 samples in the Middle Age group, and 208 samples in the Elder group (Supplementary Fig. S12a), and the PCA revealed significant heterogeneity among age groups (Supplementary Fig. S12b). Differential methylation analysis revealed a significant increase in the methylation levels of the probes for IGF2BP3 (CpG1, cg00508334, CpG2, cg17431401 and CpG3, cg20265043) located at TSS1500 (Supplementary Fig. S12c, d). To validate the methylation level of IGF2BP3 in hASCs in all age groups from infancy through adulthood to the elderly stage, methylation-specific PCR (MSP) analysis was performed on the CpG islands within the IGF2BP3 core promoter region on I-hASCs (10 samples), adult hASCs (A-hASCs; 16 samples), and E-hASCs (12 samples) (Supplementary Fig. S12e). The results indicated a progressive increase in the hypermethylation of the IGF2BP3 core promoter region with advancing age (Supplementary Fig. S12f).

The age-related changes in hypermethylation were accompanied by a successive decrease in the expression levels of IGF2BP3 and its downstream metabolic enzymes BCAT1 and GLS from I-hASCs to A-hASCs and further to E-hASCs, as illustrated in Supplementary Fig. S12g–i. Moreover, Pearson correlation analysis revealed that BCAT1 and GLS exhibited similar age-associated expression patterns to those of IGF2BP3, with a correlation coefficient (R^2^) > 0.6 and *P* < 0.001 (Supplementary Fig. S12j, k). The expression of these three molecules decreased only marginally from the I-hASCs to the A-hASCs, whereas the decreases were significantly more pronounced between A-hASCs and E-hASCs. These findings further substantiate the role of the IGF2BP3–m6A–BCAT1/GLS regulatory axis in the continuum of age-associated cellular senescence across infant, adult, and elderly cohorts.

### Glutamine and BCAA supplementation effectively restored E-hASC stemness

**Fig. 7:**
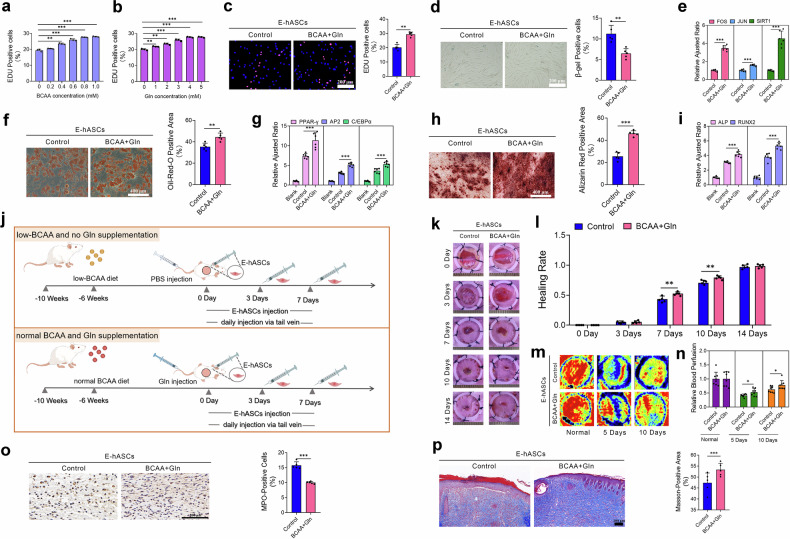
BCAA and glutamine supplementation improved the viability, lineage plasticity, and tissue remodeling potentials of E-hASCs. Quantification of EdU-positive E-hASCs after treatment with different concentrations of BCAAs (including isoleucine, leucine, and valine) (**a**) or Gln (glutamine) (**b**). **c** Representative images of EdU staining and quantification of the percentage of EdU-positive E-hASCs after BCAA and Gln supplementation. **d** SA-β-gal staining and quantification of the percentage of SA-β-gal-positive E-hASCs after BCAA and Gln supplementation. **e** Relative expression of age-related genes in E-hASCs after BCAA and Gln supplementation. **f** Oil Red O staining and quantification of positively stained areas in E-hASCs after BCAA and Gln supplementation following three weeks of adipogenic induction. **g** Relative expression of lipogenesis-related genes after adipogenic induction. **h** Alizarin Red S staining and quantification of positively stained areas in E-hASCs after BCAA and Gln supplementation following three weeks of osteogenic induction. **i** Relative expression of osteogenesis-related genes after osteogenic induction. **j** Schematic overview of the mouse model, emphasizing nutritional interventions. Representative images (**k**) and quantification of wound healing rates (**l**) at different time points after injection of E-hASCs with and without BCAA and Gln supplementation. Representative photomicrographs (**m**) and quantitative analyses (**n**) of blood perfusion in wounds after injection of E-hASCs with and without BCAA and Gln supplementation at different time points. **o** Representative images of MPO-positive cells determined by IHC staining and quantification of MPO-positive cells in wound sections after injection of E-hASCs with or without BCAA and Gln supplementation at 3 days post-surgery. **p** Representative images of Masson’s trichrome staining of wound sections after injection of E-hASCs with or without BCAA and Gln supplementation and quantification of Masson-positive areas at 14 days post-surgery. *P < 0.05; **P < 0.01; and ***P < 0.001.

Metabolic interventions have emerged as promising strategies for clinical applications, underscoring the pivotal role of metabolic regulation in cellular function and survival^33^. Therefore, we explored how glutamine and BCAA supplementation could reverse senescence and restore stemness in E-hASCs. First, E-hASC activity changed in a dose-dependent manner upon glutamine or BCAA treatment. Through systematic optimization, we determined the optimal concentrations to be 4 mM for glutamine and 0.8 mM for BCAA (Fig. 7a, b). At the indicated concentrations, supplementation with glutamine and BCAAs effectively alleviated the senescent phenotype of E-hASCs; improved their activity, proliferation, and stemness characteristics; and reduced senescence and apoptosis (Fig. 7c–i; Supplementary Fig. S13a–c).

Building on the rejuvenation potential of glutamine/BCAA supplementation in vitro, we performed nutritional interventions to explore how the metabolic microenvironment affects the therapeutic efficacy of E-hASCs in vivo. To simulate metabolic stress in vivo, we fed a custom low-BCAA diet containing one-fifth the BCAA content of a standard diet to mice^34^. The mice were fed the low-BCAA or normal diet starting at four weeks of age, and a full-thickness excisional wound model was generated at 10 weeks of age (Fig. 7j). Two hundred microliters of PBS and glutamine were subsequently injected via the tail vein of mice fed the low-BCAA or normal diet to establish distinct metabolic microenvironments for the transplanted E-hASCs (Fig. 7j). Glutamine and BCAA supplementation significantly enhanced the therapeutic efficacy of E-hASCs in promoting wound healing in vivo, accelerating tissue repair and revascularization (Fig. 7k–n). Histological analysis using MPO and Masson’s trichrome staining revealed reduced inflammatory responses on day three and increased collagen deposition on Day 14 after treatment in the wounds of mice receiving glutamine or BCAA supplementation (Fig. 7o, p). These findings underscore the promise of metabolic modulation as a translational approach to mitigate cellular aging and improve regenerative therapies.

## Discussion

hASCs have broad therapeutic potential for clinical applications, such as plastic surgery, wound healing^35^, autoimmune diseases^36^, diabetes^37^, and aging-related disorders, including osteoarthritis^38^, neurodegenerative disorders^39^, and degenerative fibrosis of the viscera^40^. However, the clinical application of hASCs is significantly hampered by functional impairment during aging, a phenomenon poorly understood despite prior reports of age-dependent reductions in MSC numbers and differentiation capacity^41^. Systematic comparison of I-hASCs and E-hASCs revealed that aging drives profound functional attrition, characterized by the accumulation of senescent cells, impaired self-renewal capacity, lineage-specific differentiation defects, and diminished tissue repair efficacy in vivo. Integrated multiomics analyses revealed a central role for metabolic reprogramming, with aged hASCs exhibiting dysregulated glutamine and BCAA metabolism and disrupted redox homeostasis. These findings improve the understanding of age-related hASC dysfunction and establish nutrient-sensitive pathways as pivotal regulators of hASC aging. This study provides a mechanistic blueprint for rejuvenating aged hASCs by linking epitranscriptomic control to metabolic vitality, offering actionable targets to enhance autologous stem cell therapy in elderly people.

Aging is a natural physiological process that occurs throughout an organism’s lifespan and is characterized by stem cell exhaustion and diminished functionality, which can compromise organ regeneration and disrupt tissue homeostasis^42^. Emerging evidence underscores the importance of metabolic plasticity and energy supply in stem cell identity and viability during aging. Previous studies have shown that mitochondrial and glucose metabolic remodeling in aged human MSCs, especially in bone marrow-derived MSCs (BMSCs), alters energy production and redox homeostasis, which significantly affect the fate of MSCs, including their self-renewal, senescence, and differentiation capacity^16,43^. Apart from glucose metabolism, glutamine and BCAAs, which also act as substrates or regulators, are essential for maintaining cellular energy metabolism, the cellular redox state, and nucleotide and amino acid biosynthesis^44,45^. During the aging process, the function of skeletal muscle stem cells (MuSCs) is disrupted because of changes in GSH metabolism. GSH ^low^ MuSCs exhibit delayed entry into S phase, decreased survival rates, impaired mitochondrial energy metabolism, and reduced regenerative capacity in vivo^15^. However, the specific regulatory mechanisms and key targets through which aging leads to the senescence and functional impairment of hASCs have not been elucidated. Our findings align with and extend this paradigm by revealing that glutamine and BCAA metabolism, orchestrated by BCAT1 and GLS, are pivotal regulators of hASC rejuvenation. Multiomics profiling demonstrated that I-hASCs exhibit increased TCA cycle, GSH synthesis, and BCAA catabolism activity, with BCAT1 and GLS acting as executors of metabolic function. Mechanistically, BCAT1 and GLS channel BCAA and glutamine-derived carbon skeletons into the TCA cycle to fuel ATP synthesis, whereas GLS-mediated glutaminolysis generates glutamate, a substrate critical for GSH production and redox homeostasis. This dual metabolic flux ensures bioenergetic competence and oxidative stress resilience, which are hallmarks of stem cell vitality. Regulating BCAA and glutamine metabolism may be a critical mechanism for maintaining the high viability and youthful state of hASCs.

hASCs constitute a heterogeneous population, and different subpopulations may exhibit varying degrees of plasticity^46^. We compared the transcriptome profiles of I-hASCs and E-hASCs to investigate cell heterogeneity and function using scRNA-seq analysis. Cluster 1 (ACTA2^+^TAGLN^+^) was predominantly detected in I-hASCs and was enriched in metabolic pathways associated with cellular anabolism and energy production. In contrast, Cluster 3 was primarily detected in the E-hASCs and was enriched in age-associated pathways that may contribute to age-related changes in hASCs. Further examination of BCAT1 and GLS distribution was conducted in the five subsets of hASCs. BCAT1 and GLS were expressed primarily in the ACTA2^+^TAGLN^+^ cell subset and are involved in various metabolic pathways crucial for maintaining normal energy metabolism and oxidative stress resistance. These findings underscore the critical role of the ACTA2^+^TAGLN^+^ cluster in sustaining hASC activity and function.

Although epigenetic dysregulation, including DNA methylation and histone acetylation, has been implicated in stem cell aging, our study revealed a previously unrecognized axis linking RNA epitranscriptomics to metabolic vitality in hASCs^47^. We demonstrated that m6A, the most prevalent post-transcriptional modification of eukaryotic RNAs, serves as a critical regulator of hASC rejuvenation through the orchestration of glutamine and BCAA metabolism^48^. Specifically, the RNA-binding protein IGF2BP3, whose expression is markedly downregulated in E-hASCs, stabilizes BCAT1 and GLS mRNAs via METTL3-mediated m6A modification. This mechanism was also reflected at the single-cell level: IGF2BP3 predominantly promoted metabolic activity in the ACTA2^+^TAGLN^+^ subpopulation of I-hASCs, sustaining mitochondrial respiration and redox homeostasis by increasing BCAA and glutamine catabolism. The age-associated decline in this axis and decrease in the proportion of the ACTA2^+^TAGLN^+^ subpopulation precipitated the collapse of the stem cell signature, as evidenced by diminished stemness, lineage plasticity, and regenerative capacity, hallmarks of hASC senescence recapitulated by IGF2BP3/METTL3 knockdown. We uniquely position the IGF2BP3-m6A-BCAT1/GLS axis as a druggable node for epitranscriptomic control and metabolic rejuvenation in aged hASCs, which connects RNA dynamics with nutrient intervention in aging. In addition, the IGF2BP3-dependent regulation of GLS, a key component of the above axis, has been independently demonstrated in glutamine metabolism in the context of endometriosis, providing convergent evidence supporting the biological significance of our proposed regulatory mechanism^49^. Moreover, we innovatively discovered that the increased methylation status of the IGF2BP3 core promoter may serve as a significant driver of the age-related decrease in IGF2BP3 expression. Although methylation analysis was conducted using whole blood samples, which reflect the overall aging state of the body, the age-associated methylation sites of IGF2BP3 identified were thoroughly validated in I-hASCs, A-hASCs, and E-hASCs. This analytical approach allows for the extrapolation of age-related methylation changes to the regulation of IGF2BP3 in other tissues and cell types, thereby substantially enhancing the generalizability of the regulation of IGF2BP3 methylation within the context of aging.

The therapeutic potential of nutrient modulation in aging has garnered increasing attention, with metabolite-targeting therapies becoming increasingly important in clinical settings to improve patient prognosis^50,51^. However, divergent perspectives persist regarding optimal dietary strategies. Restricting calories or specific nutrients, such as by inhibiting glutaminolysis and BCAA uptake, can mitigate age-related pathologies in certain contexts. However, this research demonstrated that targeted BCAA and glutamine supplementation reverses stem cell aging-related senescence by restoring metabolic–epigenetic coordination, a paradigm shift supported by robust in vitro and in vivo evidence^17,52^. Given the intricate regulatory mechanisms underlying age-related cellular senescence in hASCs, supplementation with BCAAs and glutamine was insufficient to completely restore the biological state of E-hASCs to that of I-hASCs. Nonetheless, our study comprehensively revealed the imbalance in glutamine and BCAA catabolism, which is modulated through the IGF2BP3-m6A-BCAT1/GLS axis, as a pivotal factor in the senescence of aged hASCs. More importantly, supplementation with glutamine or BCAAs presents a promising clinically applicable strategy to significantly alleviate the effects of aging.

Mechanistically, by establishing the IGF2BP3-m6A-BCAT1/GLS axis to sustain BCAA and glutamine flux, we resolved the apparent paradox between nutrient restriction and supplementation: precise tuning of metabolic pathways, rather than broad deprivation or addition, is key to the rejuvenation of hASCs. This dual intervention strategy (BCAA and glutamine supplementation) reversed aged hASC dysfunction and enhanced the therapeutic performance of these cells in wound healing models, accelerating tissue repair by reducing inflammatory cell infiltration, accelerating revascularization, and promoting collagen deposition. These findings redefine nutrient metabolism as a dynamically regulated axis in aging stem cells, where epigenetic control governs metabolic plasticity. Clinically, this approach circumvents the risks of systemic nutrient restriction by focusing on local metabolic reprogramming within transplanted hASCs, offering a safer avenue for elderly patients.

In this study, single-cell heterogeneity, m6A epitranscriptomics, and nutrient metabolism analyses were integrated to systematically elucidate the mechanism underlying hASC aging. Multiomics mapping revealed that the IGF2BP3-m6A-BCAT1/GLS axis plays a major role in sustaining glutamine and BCAA catabolism, redox balance, and energy supply in I-hASCs. This axis is therapeutically actionable: m6A modulation stabilizes BCAT1/GLS mRNAs, while nutrient supplementation restores metabolic activity, synergistically reversing aging phenotypes. scRNA-seq further revealed that the ACTA2^+^TAGLN^+^ subpopulation is a metabolically and epigenetically primed target for intervention. These insights translate into dual-target strategies involving precision RNA editing and metabolite supplementation to rejuvenate autologous hASCs. This approach leverages RNA and metabolic reprogramming to transform “discarded” aged hASCs into therapeutically potent cells ex vivo, offering an immediate protocol to optimize elderly-derived hASCs for regenerative therapies. By bridging epitranscriptomic control with metabolic plasticity, this study redefined aging as a reversible imbalance and positioned combination therapies at the forefront of regenerative medicine, offering transformative solutions for age-related tissue degeneration.

## Materials and methods

### Isolation, expansion, and identification of hASCs

Primary hASCs were isolated from discarded abdominal subcutaneous adipose tissue (SAT) from six healthy infants and six elderly individuals using 0.1% collagenase I (Sigma, St. Louis, MO, United States)^53^. Further details of the donors are provided in Supplementary Table S1. The isolated precipitate was suspended in DMEM/F12 (HyClone, United States) supplemented with 10% fetal bovine serum (FBS; Gibco, United States) and 1% penicillin/streptomycin (HyClone, United States). Primary hASCs were cultured in a T25 flask at 37 °C with 5% CO_2_. The cultured primary hASCs were harvested for further investigation, and flow cytometry was used to detect hASC surface markers, including CD29, CD44, CD90, CD105, and CD34 (Abcam, United Kingdom)^54^. When the cells reached 70%–80% confluence, they were detached with Trypsin-EDTA solution (Sigma–Aldrich) and seeded into T75 flasks at a density of 1 × 10^6^ cells/flask for expansion. Patients provided informed consent for the anonymous use of discarded SAT for research purposes, and the study was approved by the Ethics Committee of Xijing Hospital, Fourth Military Medical University (KY20202103-F-1).

### RT, amplification, and library construction for scRNA-seq

Following trypan blue staining to assess cell viability, single-cell suspensions of primary hASCs (2 × 10^5^ cells/mL) in PBS (HyClone) were loaded onto a microwell chip using the Singleron Matrix^®^ Single-Cell Processing System. Barcoding beads were collected from the microwell chip, and mRNAs captured by the barcoding beads were reverse transcribed into cDNAs, followed by PCR amplification for library construction. The scRNA-seq libraries were constructed according to the instructions provided by the GEXSCOPE^®^ Single-Cell RNA Library Kit (Singleron)^55^. The libraries were diluted to a concentration of 4 nM, pooled, and sequenced using Illumina NovaSeq 6000 with 150 bp paired-end reads.

### scRNA-seq data processing, dimensionality reduction, and clustering

Details are provided in the supplementary Materials and Methods.

### ScRNA-seq data analysis

The procedures for the cell–cell interaction analysis, TF regulatory network analysis, pseudotime analysis, cell developmental potential evaluation, and UCell gene set scoring are described in the supplementary Materials and Methods.

### Protein extraction, digestion, and labeling

Frozen primary hASCs stored in liquid nitrogen were lysed, and proteins were extracted using SDT buffer (4% SDS, 100 mM Tris-HCl, and 1 mM dithiothreitol (DTT), pH 7.6). The protein cell lysate was centrifuged at 12,000× *g* for 15 min at 4 °C to remove debris and obtain the protein supernatant. The protein concentration was quantified using a BCA protein assay kit (Bio-Rad, United States). Proteins were digested with trypsin using a filter-aided sample preparation method^56^. Afterward, the resulting peptides were desalted using an HLB column and dried under vacuum. Peptide concentrations were determined using a peptide quantification kit (Thermo Scientific, United States). Each peptide mixture of the protein sample (100 µg) was labeled with iTRAQ reagent according to the manufacturer’s instructions (Sigma–Aldrich).

### High-pH reversed-phase high-performance liquid chromatography (HPLC)

For bulk proteomic analysis, labeled peptides were separated into 24 fractions using an Acquity Ultra Performance liquid chromatograph (Waters, United States) with an Acquity UPLC BEH C18 column (1.7 µm, 2.1 × 150 mm; Waters, United States). Bound peptides were eluted from the column using a step gradient with increasing acetonitrile concentrations, starting from phase A (2% acetonitrile, pH 10) to phase B (80% acetonitrile, pH 10). Each protein sample was divided into 28 fractions and pooled to yield 14 fractions per sample. The peptides were desalted using C18 cartridges (Thermo Scientific, United States) and dried using vacuum centrifugation.

The procedures for high-resolution LC–MS/MS analysis are described in the supplementary Materials and Methods.

### Protein identification

The raw mass spectrometry (MS) proteomics data were submitted to the ProteomeXchange Consortium (http://proteomecentral.proteomexchange.org) via the iProX partner repository and assigned PXD number PXD051832. The data files for each sample were processed with MaxQuant software (version 1.6.5.0). The MASCOT server (version 2.5.1) was used to search for peak lists against the human protein databases UniProt and SwissProt. The search criteria were as follows: trypsin/P was chosen as the enzyme, with two missed cleavages allowed; carbamidomethylation on cysteine was set as a fixed modification; oxidation and N-terminal acetylation were set as variable modifications; the peptide mass tolerance was ± 20 ppm; the fragment mass tolerance was ± 20 ppm; and the false discovery rate was 1%. At least one unique peptide was identified to support protein identification. A consistency analysis of the 12 samples was performed using PCA and a sample repeatability heatmap.

### Differentially expressed genes (DEGs) and proteins (DEPs)

DEGs and DEPs between I-hASCs and E-hASCs were detected as described in the supplementary Materials and Methods.

### Pathway enrichment analysis

The procedures for pathway enrichment analysis of DEGs and DEPs in primary hASCs is described in the supplementary Materials and Methods.

### Cellular metabolomics analysis

Targeted metabolomic analysis was performed to determine the BCAA and GSH levels in primary I-hASCs and E-hASCs. The samples were redissolved in 100 μL of acetonitrile: water solution (1:1, v/v). Following centrifugation at 14,00× *g* for 15 min at 4 °C, the supernatant was subjected to LC–MS analysis. The UHPLC system was equipped with an ACQUITY UPLC BEH Amide column (100 × 2.1 mm, 1.7 μm). Separation was conducted using a linear gradient of mobile phases A (90% water, 2 mM ammonium formate, and 10% acetonitrile) and B (0.4% formic acid in acetonitrile) at a flow rate of 300 μL/min. Mass spectrometric data were acquired using a UHPLC system (Agilent Technologies, United States) coupled to a QTRAP^®^ 6500+ mass spectrometer (ABSciex, United States) equipped with an electrospray ionization (ESI) source operating in positive (ESI + ) and negative (ESI–)modes.

### Metabolomics data processing

The procedures for the targeted metabolomics analysis are described in the supplementary Materials and Methods.

### DNA methylation analysis

The procedures for the DNA methylation analysis are described in the Supplementary Materials and Methods.

### EdU staining assay

EdU staining of hASCs was conducted using an EdU staining kit (Beyotime, China) according to the manufacturer’s instructions. Cell proliferation was assessed by computing the percentages of EdU^+^ cells.

### Senescence-associated β-galactosidase (SA-β-Gal) staining assay

The hASCs were stained with SA-β-gal to detect cellular senescence. SA-β-gal staining was performed using a β-galactosidase staining kit (Beyotime, China) according to the manufacturer’s instructions. The percentage of β-gal-positive cells among the total number of hASCs was calculated for each group.

### Calcein-AM/PI staining

hASC viability was assessed using a calcein-AM/PI double-staining kit (Beyotime, China). I-hASCs and E-hASCs were inoculated in a 24-well plate at a density of 2 × 10^4^/well. Cell morphology and viable cell counts per unit area were determined after 12 h and 48 h.

### Cell migration assay

Cell migration was measured using a scratch assay^57^. At 0 and 24 h after scratch establishment, cell migration was recorded using a microscope, and the scratch area was quantified using ImageJ software. Migration rate = [(0 h scratch area − 24 h nonmigrated area)/0 h scratch area] × 100%.

### hASC differentiation assays

Adipogenesis and osteogenesis were induced in hASCs using osteogenic and adipogenic induction/maintenance media (Longza, United States)^58^. After three weeks of osteogenic and adipogenic induction, the differentiation of hASCs was evaluated using Alizarin Red S (Sigma, United States) and Oil Red O staining (Sigma, United States), respectively. The percentages of Oil Red O- and Alizarin Red S-positive areas of the total areas were quantified using ImageJ software from 5 different randomly selected regions for each group.

### Transfection and RNA interference

RNA overexpression plasmids and short hairpin RNAs (shRNAs) were synthesized by Qingke Biotechnology (Beijing, China). Plasmid and shRNA transfection assays were conducted with hASCs as previously described^59,60^. The sequences of two shRNA duplexes targeting IGF2BP3, METTL3, BCAT1, and GLS are listed in Supplementary Table S4. Control shRNA (NC), control plasmid (pcDNA), shIGF2BP3#1 (shIGF2BP3), shMETTL3#1 (shMETTL3), an IGF2BP3 overexpression plasmid (OE-IGF2BP3), a BCAT1 overexpression plasmid (OE-BCAT1), or a GLS expression plasmid (OE-GLS) were used to transfect hASCs for functional experiments.

### Generation of IGF2BP3-KO cell lines

Immortalized hASCs (ADHX-C1061 cells; Lot Number: 241213Y302) obtained from HyCyte™ (Suzhou, China) were utilized to generate IGF2BP3-KO cell lines. hASC immortalization was achieved by cotransfecting them with Hygro-CMV-hTERT and pLV-Puro-CMV-SV40 lentiviral particles following the manufacturer’s instructions. The primer sequences used and the expression levels of SV40 and hTERT in ADHX-C1061 cells are presented in Supplementary Table S2 and Fig. S14a, b, respectively. The cellular characteristics, including morphology, osteogenic, adipogenic, and chondrogenic differentiation capacity; and surface marker expression profiles (CD73^+^, CD105^+^, CD90^+^, CD45^–^, CD11b^–^, and CD34^–^), as provided in the quality inspection certificate of ADHX-C1061, are illustrated in Supplementary Fig. S14c–g. Single guide RNAs (sgRNAs) targeting IGF2BP3 were designed using an online CRISPR tool (https://crispr.dbcls.jp), with the specific target sequences sg-IGF2BP3-1-ATATCCCGCCTCATTTACAG and sg-IGF2BP3-2-TGATTTGCCTCTGCGCCTGC^61^. The CRISPR/Cas9-sgRNA construct targeting IGF2BP3 was integrated into a CRISPR/Cas9 lentiviral plasmid and transfected into the ADHX-C1061 cell line. Subsequently, puromycin-resistant clones were isolated, and their knockdown efficiency was rigorously tested through western blot analysis.

### RNA extraction and qRT–PCR

Total cellular RNA was extracted using TRIzol reagent (Invitrogen, United States), and cDNA was obtained via reverse transcription using PrimeScript RT Master Mix (TaKaRa Biotechnology, China) according to the manufacturer’s guidelines. The expression of target genes was determined using RT–qPCR with SYBR-Green qPCR master mix (TaKaRa Biotechnology, China). GAPDH was used to normalize gene expression in the cell samples, and the relative expression of target genes was calculated using the 2^−ΔΔCt^ method. The primer sequences used are presented in Supplementary Table S2.

### Western blotting assay

HASCs were lysed, and proteins were extracted using RIPA buffer (Beyotime, China) supplemented with EDTA-free protease inhibitor cocktail (Thermo Scientific, United States). A BCA protein assay kit (Thermo Scientific, United States) was used to determine the protein concentrations. Denatured proteins were separated using 10% SDS–polyacrylamide gel electrophoresis (SDS–PAGE) and transferred to PVDF membranes. The membranes were blocked with skim milk and incubated with primary antibodies, followed by incubation with horseradish peroxidase (HRP)-labeled secondary antibodies (ZSGB-BIO; Beijing, China) for 1 h. Immunoreactivity was detected using an enhanced chemiluminescence (ECL) kit (Merck Millipore, Billerica, MA, United States), and β-actin was used as a loading control. The primary antibodies used were anti-IGF2BP3 (#57145; 1:1000; Cell Signaling Technology), anti-BCAT1 (#88785; 1:1000; Cell Signaling Technology), and anti-GLS (#56750; 1:1000; Cell Signaling Technology) antibodies. Blots were imaged using an Odyssey infrared imaging system (LI-COR Biosciences, United States).

### BCAT1 and GLS enzyme activity assays

Recombinant human BCAT1 and IGF2BP3 proteins were obtained from Proteintech (Wuhan, China), and recombinant bovine GLUD1 was obtained from Sigma–Aldrich (USA). The BCAT1 activity assay was performed in 96-well plates, with each well containing 200 μL of 100 mM Tris buffer (pH 7.4) supplemented with 50 mM pyridoxal 5’-phosphate, 5 mM NAD⁺, 5 mM DTT, 5 mM L-leucine, 100 μg of GLUD1, and 50–250 ng of recombinant transaminase. IGF2BP3 was incorporated into the reaction mixture of the designated wells. Kinetic spectrophotometric analysis was conducted by monitoring the absorbance at 340 nm (A340) to measure the rate of NADH formation. The GLS activity assay kit was obtained from Abcam (USA), and the assay was performed in strict accordance with the manufacturer’s guidelines.

### Glutamine and BCAA uptake assays

hASCs (1 × 10^5^) were cultured in six-well plates until they reached 80%–90% confluence, and the glutamine and BCAA uptake were determined by subtracting the glutamine and BCAA concentrations in the medium after 24 h of incubation from the concentrations in plates without hASCs (control plates). The concentrations of BCAAs and glutamine in the culture medium were determined using a BCAA detection kit (MAK003; Sigma) and a glutamine kit (ab197011; Abcam), following the manufacturers’ instructions. Relative optical density (OD) values were measured using a microplate reader.

### Intracellular GSH, glutamate, succinate, fumarate, and α-KG measurements

Glutamine and BCAA metabolism in hASCs were assessed by calculating the intracellular GSH, glutamate, succinate, fumarate, and α-KG levels after 24 h of incubation in six-well plates with complete medium. Intracellular GSH, glutamate, succinate, fumarate, and α-KG levels were determined using GSH (#ab205811; Abcam), glutamate (#ab83389; Abcam), succinate (#ab204718; Abcam), fumarate (#ab102516; Abcam), and α-KG (#ab83431; Abcam) assay kits, respectively, according to the manufacturer’s protocol. The absorbance measured at 450 nm was used to calculate the concentrations of the metabolites.

### ROS assay

A ROS detection kit (Beyotime Biotechnology) was used to identify differences in ROS production between I-hASCs and E-hASCs. Following a 24-h incubation in six-well plates, hASCs were pretreated with 5 mM hydrogen peroxide (H_2_O_2_) for 30 min to induce ROS production. Afterward, the hASCs were harvested and analyzed using a flow cytometer with excitation at 488 nm and detection at 525 nm.

### Seahorse assay

The OCR in hASCs was measured using Seahorse XFe24 Flux Analyzer (Agilent Technologies, Santa Clara, California) according to the manufacturer’s instructions^62^. Briefly, hASCs were seeded onto collagen-pre-coated Seahorse XFe24 well plates at a density of 20,000 cells/well overnight. Afterward, the basal OCR was measured, followed by sequential treatment with 1 μM oligomycin A, 1 μM carbonyl cyanide 4-(trifluoromethoxy) phenylhydrazone, and 1 μM rotenone/antimycin A. Each experimental group consisted of three replicates, the values were normalized to the number of cells in each well at the end of the assay, and the data were analyzed using Seahorse Wave software.

### Luciferase reporter assay

Luciferase reporter assays were performed as previously described^63^. The dual luciferase assay was performed using a Firefly Renilla luciferase assay kit (MedChemExpress, United States). A 492-nt WT sequence containing the GLS + 4376 site and a 456-nt WT sequence containing the BCAT1 + 1221 site were synthesized, cloned, and inserted into the pMIR-REPORT vector (Ambion). The quick change site-directed mutagenesis technique was employed to mutate the m6A modification sites of BCAT1 and GLS. I-hASCs with METTL3 knockdown (shMETTL3) were plated at a density of 1 × 10^5^ cells/well in 24-well plates. Luciferase reporter plasmids (100 ng) and pRL-TK Renilla luciferase plasmids (20 ng) were cotransfected with or without IGF2BP3 expression vectors using Lipofectamine 3000 (Invitrogen).

### RNA stability assay

After 24 h of incubation, hASCs (1 × 10^5^) were plated into six-well plates and exposed to 5 μg/mL actinomycin D (MedChemExpress, United States). The hASCs were collected at specific time points for RNA isolation and RT–qPCR analysis.

### RNA m6A dot-blot assay

An m6A dot-blot assay was performed to investigate global mRNA levels as previously described^64^. The indicated total RNA of hASCs was sufficiently mixed and denatured in a threefold volume of RNA incubation buffer at 65 °C for 5 min. Then, the RNS solution was maintained by the addition of 20× saline-sodium citrate solution (Sigma–Aldrich, United States) at 4 °C. Fractioned RNA was transferred to an Amersham Hybond-N+ membrane (Cytiva, China) using a Bio-Dot apparatus (Bio-Rad, United States). Following ultraviolet cross-linking, the membrane was stained with 0.02% methylene blue for 5 min. Then, the membrane was washed with 1× PBST and blocked with 5% nonfat milk, followed by incubation with an anti-m6A antibody (1:1000, #CS220007, Millipore) overnight at 4 °C. Afterward, the membrane was incubated with an HRP-conjugated secondary antibody (Santa Cruz, United States). The membrane was visualized using chemiluminescence with Pierce ECL western blotting substrate (Thermo Fisher Scientific, United States).

### RIP

The RIP assay was conducted using the PureBinding^®^ RNA Immunoprecipitation Kit (Geneseed) following the manufacturer’s instructions. IGF2BP3 antibody-bound chromatin was captured using protein A/G magnetic beads at room temperature for 30 min. Subsequently, the beads were washed three times with RIP buffer and incubated with precleared nuclear extracts at 4 °C overnight. Total RNA was used as the input control for RT–qPCR analysis of the extracted RNA. U1 was used as a negative control. Each RIP assay was conducted in triplicate using three biological replicates. The primers used are listed in Supplementary Table S3.

### MeRIP–qPCR

m6A modification of BCAT1 and GLS was determined using the Magna MeRIP m6A Kit (Millipore) according to the manufacturer’s protocol. Briefly, 5 μg of an anti-m6A antibody (#CS220007, Millipore) or normal mouse IgG (#CS200621, Millipore) was prewashed and incubated with Magna ChIP protein A/G magnetic beads (#CS203152, Millipore) for 30 min at room temperature. Afterward, the antibody-bead mixtures were combined with purified poly(A) RNA, and qRT–PCR was conducted to measure the enrichment of m6A-containing mRNA using the primers listed in Supplementary Table S3.

### RNA pull-down assay

S1m-tagged BCAT1 and GLS were constructed as previously described^65^. The WT or Mut constructs were transfected into hASCs plated in 10 cm dishes. hASCs were harvested and lysed in 1.5 mL of lysis buffer containing protease inhibitors (Roche) 48 h posttransfection. After centrifugation at 4 °C for 20 min, the supernatant was collected, with 10% reserved as the input control. The remaining lysate was incubated with streptavidin beads for 4 h at 4 °C. The purified pellet was resuspended in 40 μL of 2× SDS–PAGE sample buffer and subjected to western blotting using anti-BCAT1 and anti-GLS antibodies.

### Analysis of methylation sites

Data on IGF2BP3 gene methylation were downloaded from the Xena browser (https://xenabrowser.net). The illuminaMethyl450_hg38_GDC text was downloaded from the GEO database (https://www.ncbi.nlm.nih.gov/geo/query/acc.cgi). R was used to investigate the genomic position, including the promoter transcription start site (TSS) and gene body, and correlation analysis between gene expression and methylation level was performed. Finally, lollipop diagrams were generated using the trackViewer package in R software.

### Supplementation of E-hASCs with BCAAs and glutamine

Primary E-hASCs were incubated for 24 h with different concentrations of BCAAs (Leu, Val, and Ile; 0–1 mM) and glutamine (0–5 mM) in complete DMEM/F12, and EdU assays were used to determine the most appropriate concentrations of BCAA and glutamine to restore viability. Afterward, 0.8 mM Leu, 0.8 mM Val, 0.8 mM Ile, and 4 mM glutamine were added to the complete medium to reverse the aging-related features of E-hASCs.

### Animals and wound model

The animal procedures were approved by the Fourth Military Medical University Medical Ethics Committee (IACUC20241266). A murine full-thickness skin defect wound model (diameter: 10 mm) was established in BALB/c mice (eight weeks old, female) to determine the effect of hASCs on wound healing. Wounds were stented with silicone rings with inner and outer diameters of 10 and 15 mm, respectively, and sutured to the skin with 6–0 nylon to prevent wound contraction, as previously described^66^. I-hASCs and E-hASCs (5 × 10^5^ cells in 200 µL of PBS) at passage four or PBS (200 µL) were transplanted into healthy tissue at the wound boundary by multiple-point injection at 0, 3, and 7 days after full-thickness skin excision. The wound areas were photographed on Days 0, 3, 7, 10, and 14 after skin excision and measured using ImageJ software. Wound healing rate = [(initial wound area-current wound area)/initial wound area] × 100%. Superficial blood flow in the wound was detected using laser Doppler flowmetry (Perimed AB, Jarfalla, Sweden) before and after skin excision at the indicated time points (normal skin and 3 and 10 days after surgery). Neutrophil recruitment, an index of the inflammatory state, was evaluated by immunohistochemical staining for MPO using an anti-MPO antibody (Abcam, United States, dilution: 1:100) and is expressed as the percentage of neutrophils that were cytoplasmically positive for MPO at three days post-surgery. Masson’s trichrome staining was performed using a Masson’s trichrome kit (Solarbio, China) to detect collagen deposition in the wound at 14 days post-surgery, and collagen deposition was analyzed using ImageJ software.

### Fat transplantation model

Fat was harvested from healthy adults for fat injection using the Coleman technique^67^. The obtained adipose granules were injected subcutaneously into nude mice using a 1-mL syringe with a 17-gauge needle at three locations (0.3 mL or 0.295 g at each location). To determine the effects of I-hASCs and E-hASCs on fat retention after transplantation, I-hASCs or E-hASCs (5 × 10^6^, fourth passage) in 0.2 mL of PBS were premixed with human adipose granules, and the wounds were sutured with 6–0 nylon after injection. Two months after fat transplantation, the grafts were harvested and weighed, with 5 grafts in each group for statistical analysis. The slides were stained with H&E. The integrity of the adipose tissue, inflammatory cell infiltration in the tissue, the presence of cysts and vacuoles, and the degree of fibrosis were evaluated histologically using the methods of Guo et al.^68^. These parameters were graded on a scale ranging from 0 to 5 as follows: 0, absence; 1, minimal presence; 2, minimal to moderate presence; 3, moderate presence; 4, moderate to extensive presence; and 5, extensive presence. All evaluations were performed by two researchers (senior doctors) who were blinded to sample identification.

### In vivo fluorescence imaging

Lentiviral vectors expressing mCherry were produced by Qingke Biotechnology (Beijing, China) and transfected into both I-hASCs and E-hASCs. mCherry-labeled hASCs were subsequently used to treat full-thickness skin defects and fat transplantation models as described above. For the full-thickness skin defect model in BALB/c mice, mCherry-labeled hASCs were injected at the wound boundary via the multiple-point injection method, and the spatial distributions of cellular fluorescence were assessed at 2, 24, 48, 72, 96, and 120 hours post-injection. In the fat transplantation nude mouse model, a mixture of mCherry-labeled hASCs and human adipose granules was injected subcutaneously, and the fluorescence signal distributions of the mixture were evaluated at 0, 1, 2, 3, 4, 5, 6, 7, and 8 weeks after subcutaneous transplantation. In vivo fluorescence imaging was conducted using the IVIS Lumina system (PerkinElmer, USA) with an excitation wavelength of 587 nm. Subsequent image analyses were performed utilizing Living Image software version 4.3.1 (Caliper Life Sciences, USA).

### Low-BCAA diet and glutamine supplementation in mice

BALB/c mice were randomly divided into two groups: a normal diet and a low-BCAA diet group, and fed a normal (1× BCAA) or low-BCAA (1/5× BCAA) diet for six weeks after being fed a normal diet for four weeks. Afterward, a full-thickness skin defect wound model was constructed. After skin excision, PBS (200 µL) was administered via the tail vein in the low-BCAA diet group, while 673 mg/kg glutamine dissolved in PBS (200 µL) was administered via the tail vein in the normal diet group for glutamine supplementation.

## Supplementary information

Supplementary Information

Informed Consent

## Data Availability

All data that support the findings of this study are available from the corresponding authors upon reasonable request. The gene expression profile by microarray in this paper has been deposited in NCBI GEO: GSE267783. The core programming codes used in this paper have been documented in the GitHub repository, with the link of https://github.com/ZhEnGhAOOO/programming-code/tree/main.
