# Supplementary material for: IGF2BP3-dependent glutamine/BCAA metabolic rewiring rejuvenates aged human adipose-derived stem cells for enhanced tissue regeneration
Source: Cell Discov. 2026 Jan 20;12:5. doi: 10.1038/s41421-025-00860-7 (PMC12819398; doi:10.1038/s41421-025-00860-7)
Supplement: Supplementary file 1 — Supplementary Information [file 41421_2025_860_MOESM1_ESM.pdf]

Supplementary Figures and Figure Legends

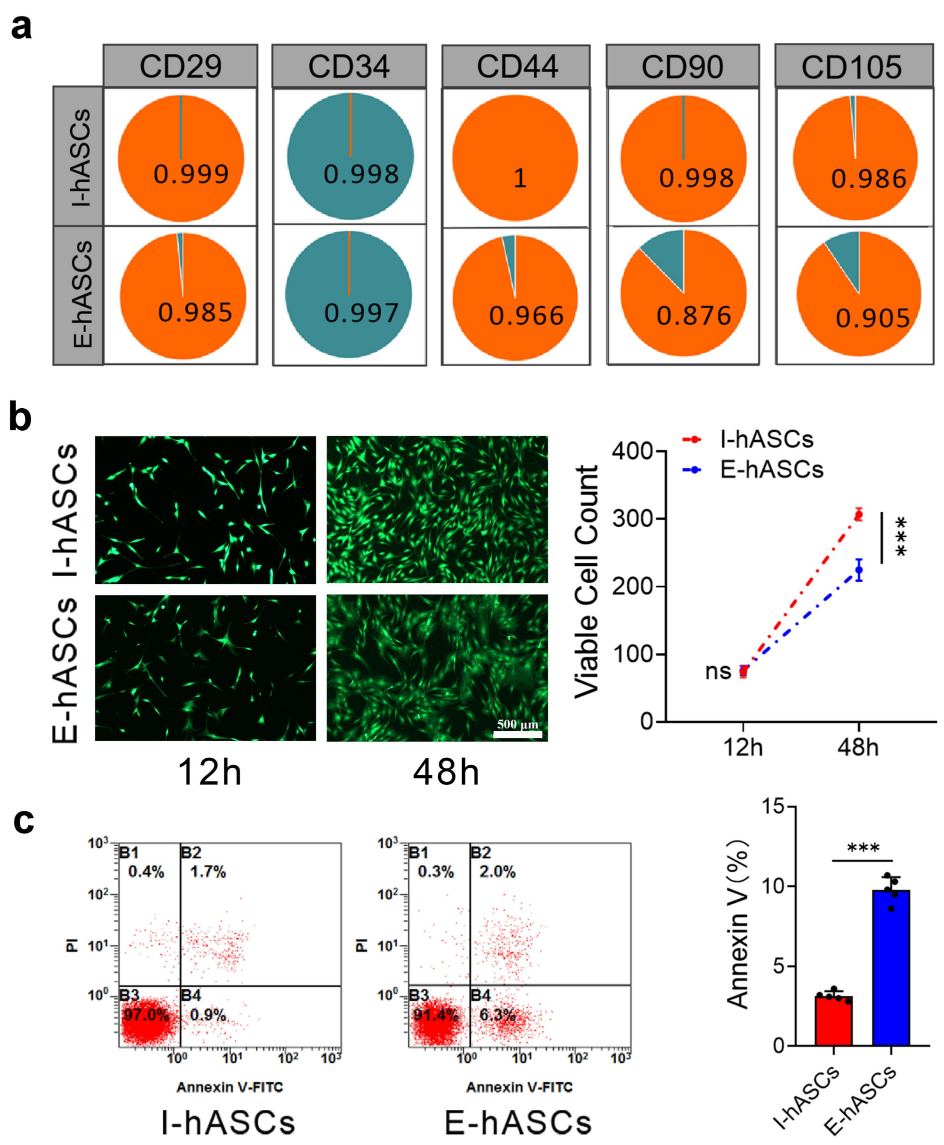

Supplementary Fig. S1. Age-related alterations in primary hASCs obtained from different ages.

a. Expression of surface markers CD29, CD34, CD44, CD90, and CD109 in primary I-hASCs and E-hASCs. b. Representative images of calcein-AM/PI staining and quantification of viable cells at 12 and 48 h for primary I-hASCs and E-hASCs. c. Apoptosis analysis and quantification of apoptotic cells in primary I-hASCs and E-hASCs. * * * represent *P*-value < 0.001.

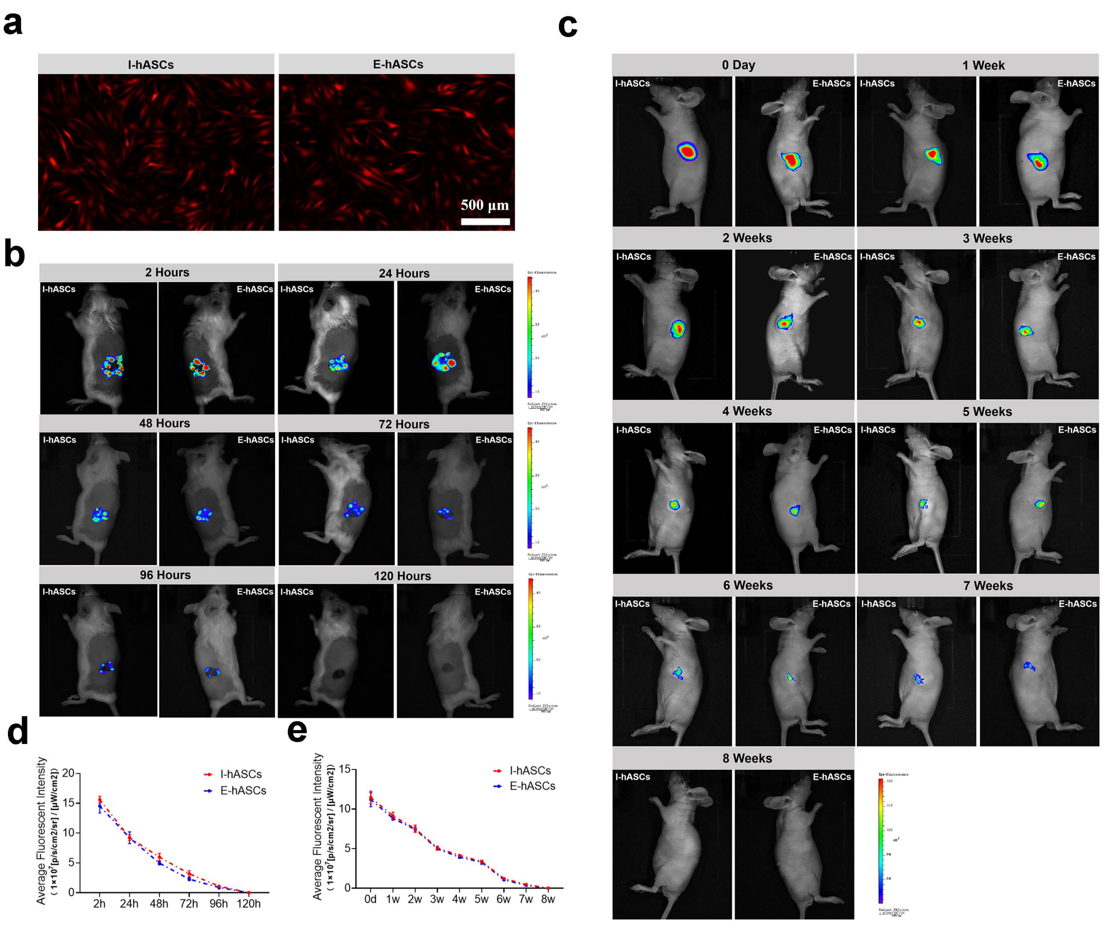

Supplementary Fig. S2. The distribution, migration, and survival duration of hASCs in vivo model.

a. Representative images of mCherry-labeled I-hASCs and E-hASCs in vitro. b. Representative images of distribution, migration, and survival duration of mCherry-labeled I-hASCs (left) and E-hASCs (right) at equivalent fluorescent intensities at indicated time ponits following the injection of mCherry-labeled hASCs at the wound boundary. c. Representative images of distribution, migration, and survival duration of mCherry-labeled I-hASCs (left) and E-hASCs (right) at equivalent fluorescent intensities at indicated time ponits following the subcutaneous injection of a mixture of mCherry-labeled hASCs and human adipose granules. d-e. Quantifications of the average mCherry-fluorescent intensities in wounds (d) and fat grafts (e) at indicated time points provided the estimates of the relative survival durations of the hASCs in wounds and fat grafts, respectively.

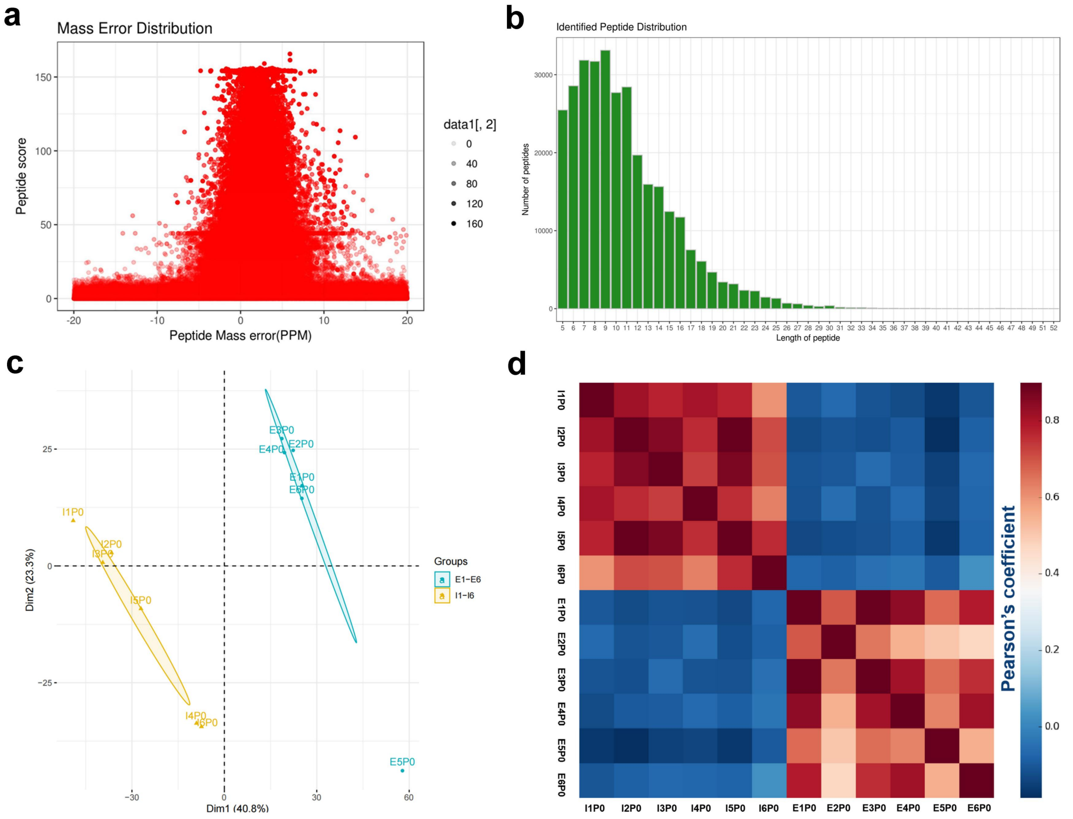

Supplementary Fig. S3. LC-MS/MS analysis of primary I-hASCs and E-hASCs.

a. Mass error distribution of all identified peptides in primary hASCs. b. Peptide length distribution. c. Principal component analysis was performed on the proteomics data from the 12 samples. d. Sample repeatability based on LC-MS/MS analysis.

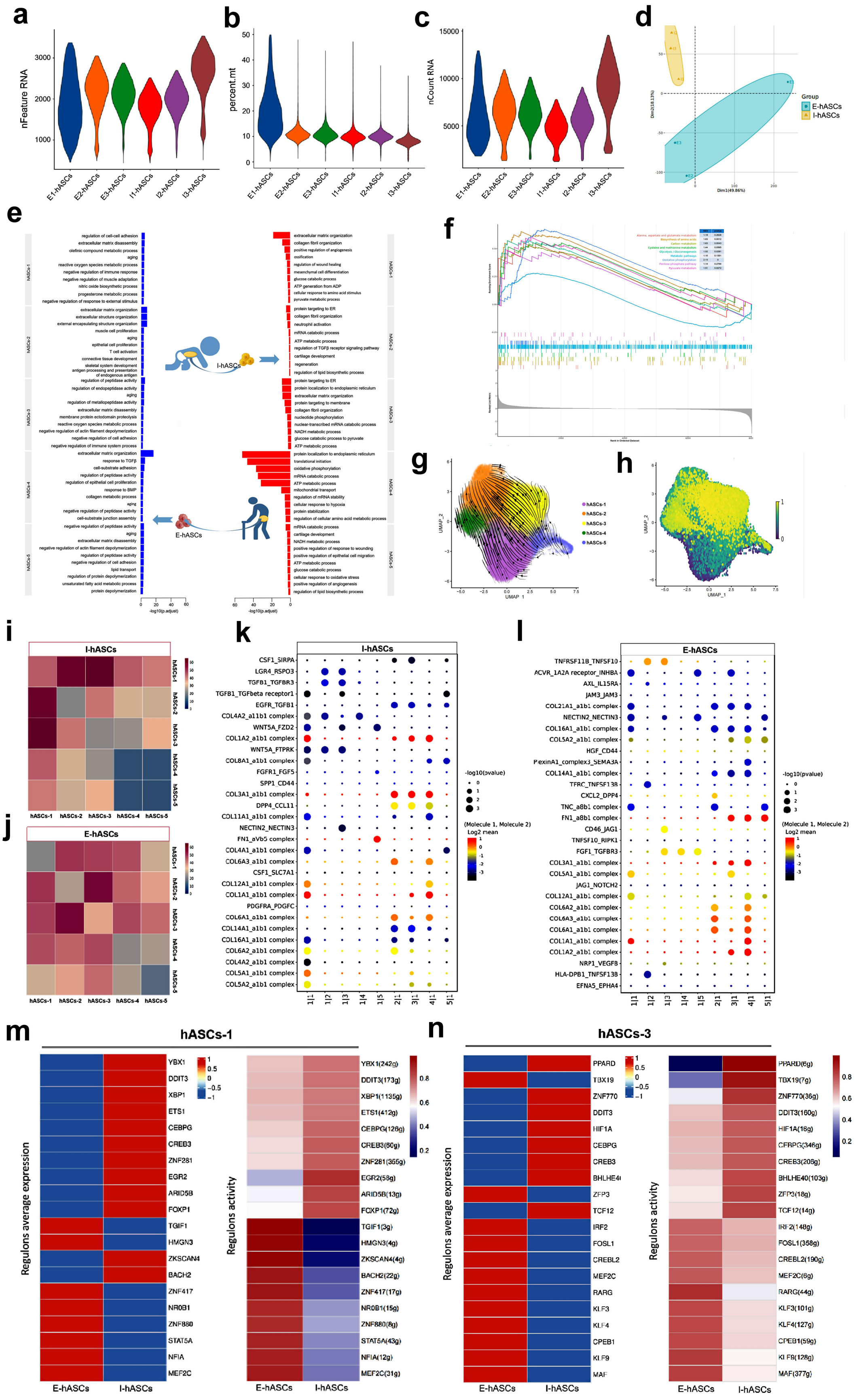

Supplementary Fig. S4. The scRNA-seq analysis of primary I-hASCs and E-hASCs.

a-c. Violin plots for each sample depict the number of RNA features (nFeature RNA) (a), absolute numerical count of RNA (nCount RNA) (b), and percent mitochondria (percent.mt) (c). d. Principal component analysis was performed on scRNA-seq data from six samples. e. In each cell cluster, the significantly enriched GO-BP pathways are depicted with red columns for I-hASCs and blue columns for E-hASCs. f. GSEA revealed differences in the enrichment of metabolic pathways between cluster 1 (ACTA2+TAGLN+) and other cell clusters in primary hASCs, with the normalized enrichment score (NES) and p-value statistics displayed in the upper right panel. $|NES| > 1$ and P-value < 0.05 were considered to indicate statistical significance. g. The scVelo velocities projected on the UMAP embedding in 5 cell subpopulations and shown as streamlines. h. The latent time featureplot confirmed the emergence of hASCs. The values ranging from 0 to 1 in the latent time featureplot represented the progression from early to late time points in cell generation, with 0 indicating early and 1 indicating late stages. i-j. The number of ligand-receptor pairs among the five cell clusters in I-hASCs (i) and E-hASCs (j) are displayed in heatmaps. k-l. The top 30 ligand-receptor pairs between cluster 1 and the other four cell clusters in I-hASCs (k) and E-hASCs (l) are presented in bubble plots. m. Heatmaps displaying the average expression (left) and activity (right) of the top 10 regulons inferred by PySCENIC analysis of I-hASCs and E-hASCs in cluster 1 of primary hASCs. n. Heatmaps displaying the average expression (left) and activity (right) of the top 10 regulons inferred by PySCENIC analysis of I-hASCs and E-hASCs in cluster 3 of primary hASCs.

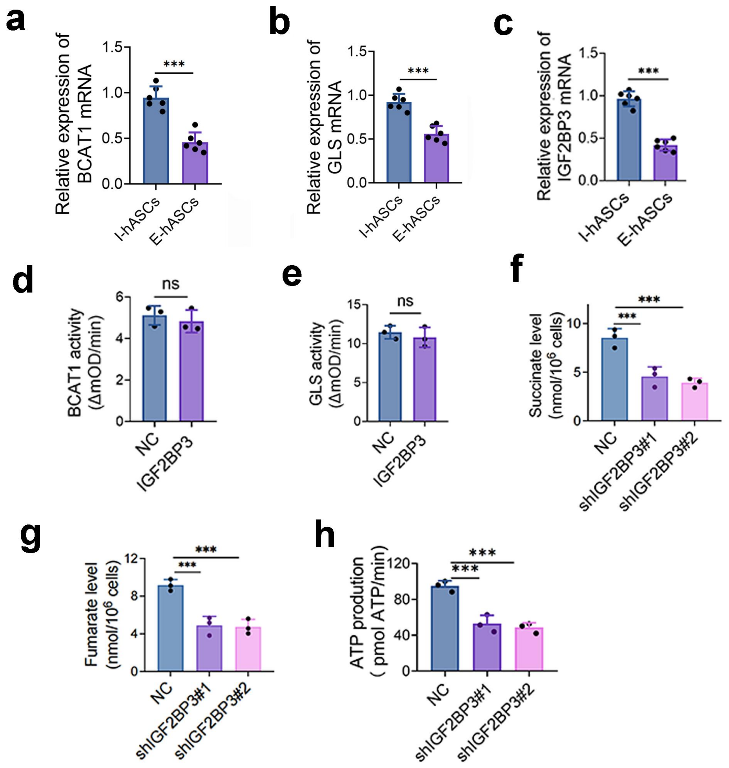

Supplementary Fig. S5. Alterations in metabolism, enzyme activity, and the expression of downstream metabolic enzymes of IGF2BP3.

a-c. Differential expression of BCAT1 (a), GLS (b) and IGF2BP3 (c) mRNA between primary I-hASCs and E-hASCs detected using qRT-PCR. d-e. BCAT1 (d) and GLS (e) enzyme activities were detected in vitro with or without IGF2BP3 protein in the reaction mixture. f-h. Levels of succinate (f), fumarate (g), and ATP production (h) in I-hASCs transfected with shIGF2BP3#1 and shIGF2BP3#2. * * * represent *P*-value < 0.001.

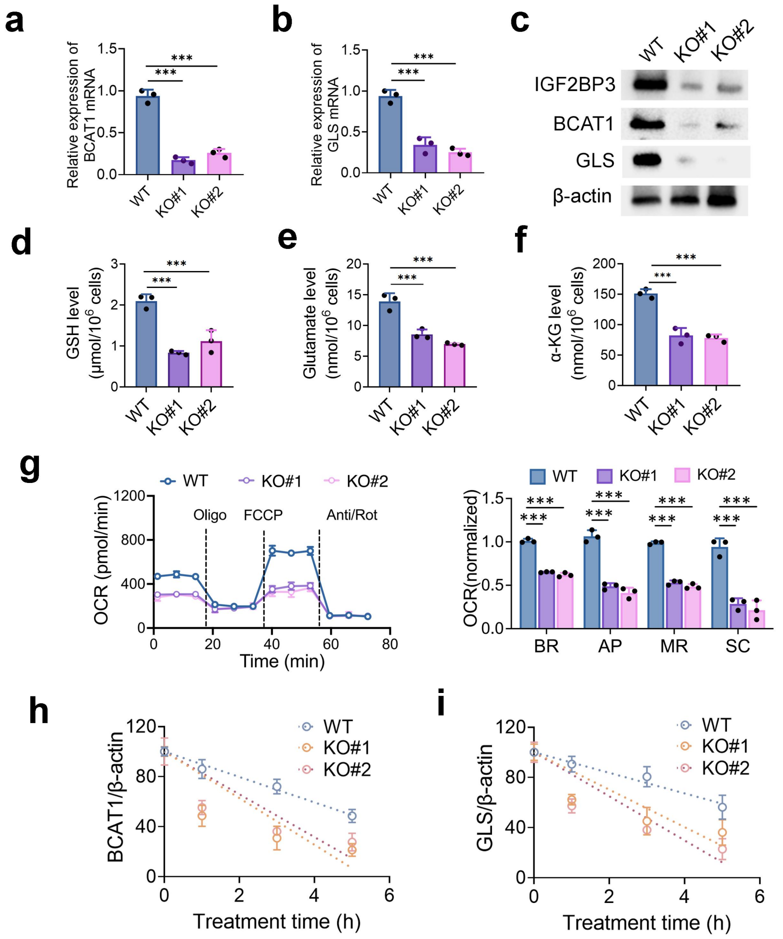

Supplementary Fig. S6. The characteristics of IGF2BP3-KO hASCs.

a-b. Differential expression of BCAT1 (a) and GLS (b) mRNA among wild-type (WT) and IGF2BP3-KO (KO #1 and #2) hASCs detected by qRT-PCR. c. Western blotting was used to detect the expression of IGF2BP3, BCAT1, and GLS in wild-type (WT) and IGF2BP3-KO (KO #1 and #2) hASCs. d-f. Levels of GSH (d), Glutamate (e), and α-KG (f) in wild-type (WT) and IGF2BP3-KO (KO #1 and #2) hASCs. g. The OCRs of wild-type (WT) and IGF2BP3-KO (KO #1 and #2) hASCs, and BR, AP, MR, and SC quantifications in each group. h-i. Stability of BCAT1 (h) and GLS (i) mRNA in wild-type (WT) and IGF2BP3-KO (KO #1 and #2) hASCs. * * * represent P-value < 0.001.

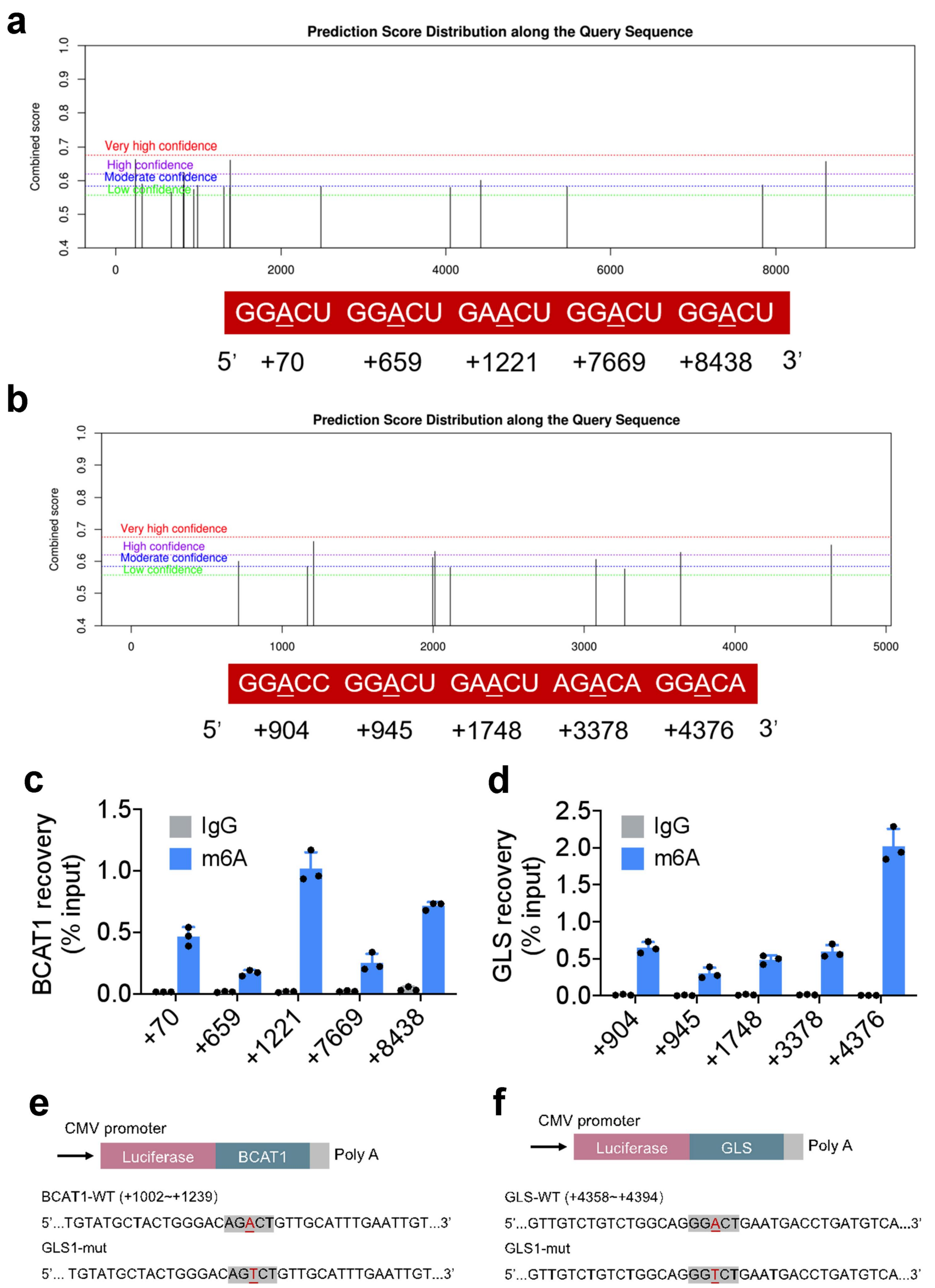

Supplementary Fig. S7. IGF2BP3 regulated the BCAT1 and GLS expression level in an m6A-dependent manner.

a-b. The graphics illustrate the five high-confidence m6A sites predicted by SRAMP in BCAT1 (a) and GLS (b) mRNA, with nucleotide positions numbered concerning the translational start sites of BCAT1 and GLS. c-d. MeRIP assay analysis of BCAT1 (c) and GLS (d) mRNA retrieved using an m6A antibody at the five high-confidence m6A sites in primary hASCs. e-f. Schematic diagrams of BCAT1-WT, BCAT1-mut (e), and GLS-WT, GLS-mut (f) firefly luciferase reporters. The 456-nt DNA sequence of WT BCAT1 and 492-nt sequence of WT GLS were inserted at the XhoI site ahead of the stop codon of the firefly luciferase gene in a pMIR-REPORT vector to generate BCAT1-WT and GLS-WT reporters, respectively. For BCAT1-mut and GLS-mut reporters, A-T substitutions (presented in red) were made within the m6A consensus site (in grey background).

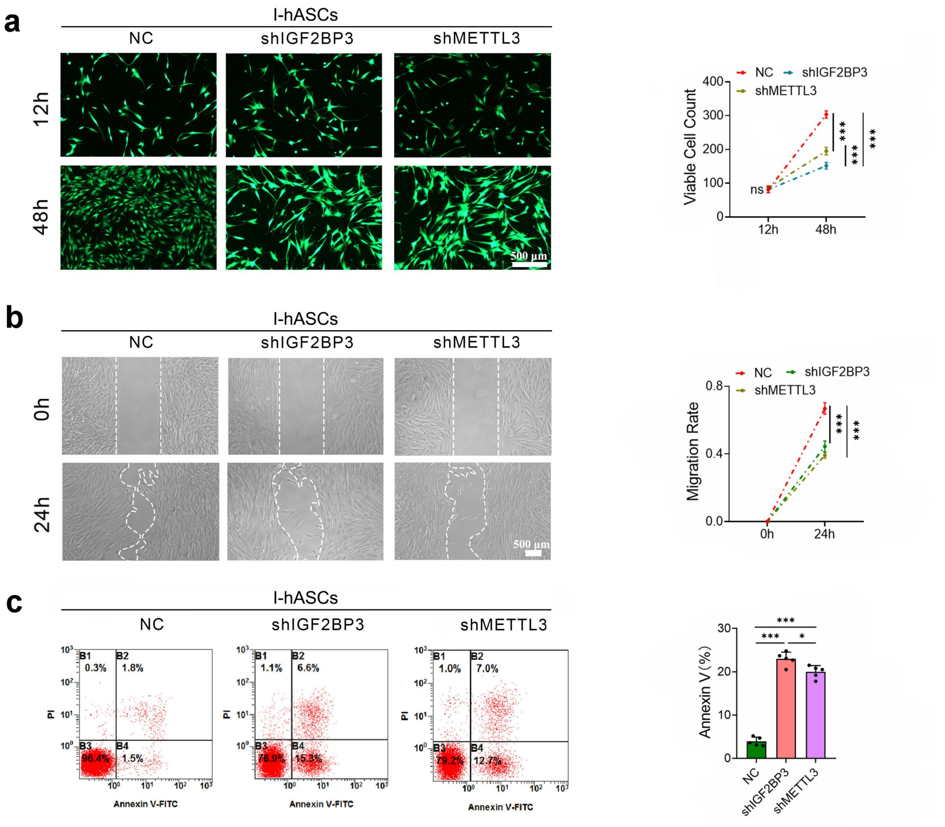

Supplementary Fig. S8. Downregulation of IGF2BP3 or METTL3 impaires the cell viability in I-hASCs.

a. Representative images of calcein-AM/PI staining and the quantification of viable cells after 12 and 48 h for I-hASCs with shIGF2BP3#1 or shMETTL3#1 transfection. b. Representative images of scratch assay and quantification of migration rates after 24 h for I-hASCs transfected with shIGF2BP3#1 or shMETTL3#1. c. Apoptosis analysis and the quantification of apoptotic cells for I-hASCs with shIGF2BP3#1 or shMETTL3#1 transfection. * represent *P*-value < 0.05; * * * represent *P*-value < 0.001.

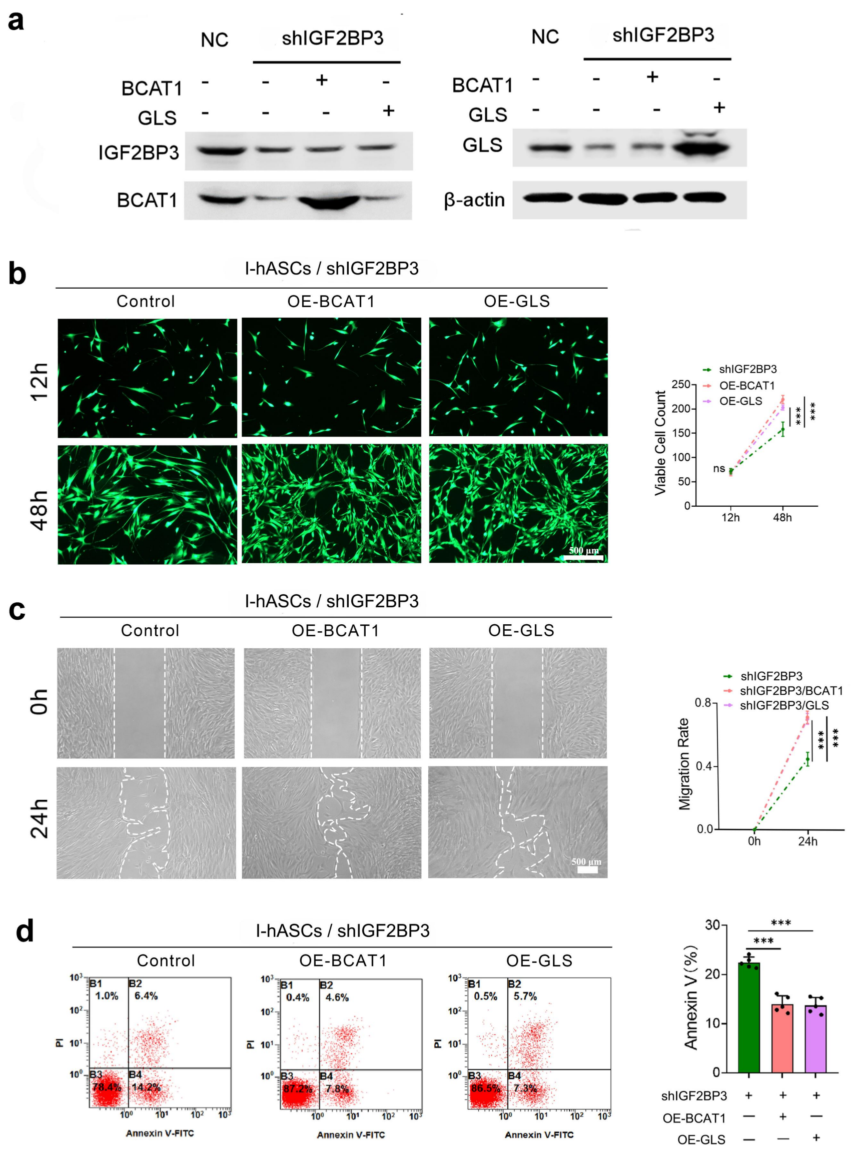

Supplementary Fig. S9. BCAT1 and GLS overexpression rescued cell viability in shIGF2BP3 transfected I-hASCs.

a. Western blotting was used to detect the expression of IGF2BP3, BCAT1, and GLS in shIGF2BP3#1 transfected I-hASCs after BCAT1 or GLS overexpression. b. Representative images of calcein-AM/PI staining and quantification of viable cells after 12 and 48 h for shIGF2BP3#1 transfected I-hASCs after BCAT1 or GLS overexpression. c. Representative images of scratch assay and quantification of migration rates after 24 h for shIGF2BP3#1 transfected I-hASCs after BCAT1 or GLS overexpression. d. Apoptosis analysis and quantification of apoptotic cells for shIGF2BP3#1 transfected I-hASCs after BCAT1 or GLS overexpression. * * * represent *P*-value < 0.001.

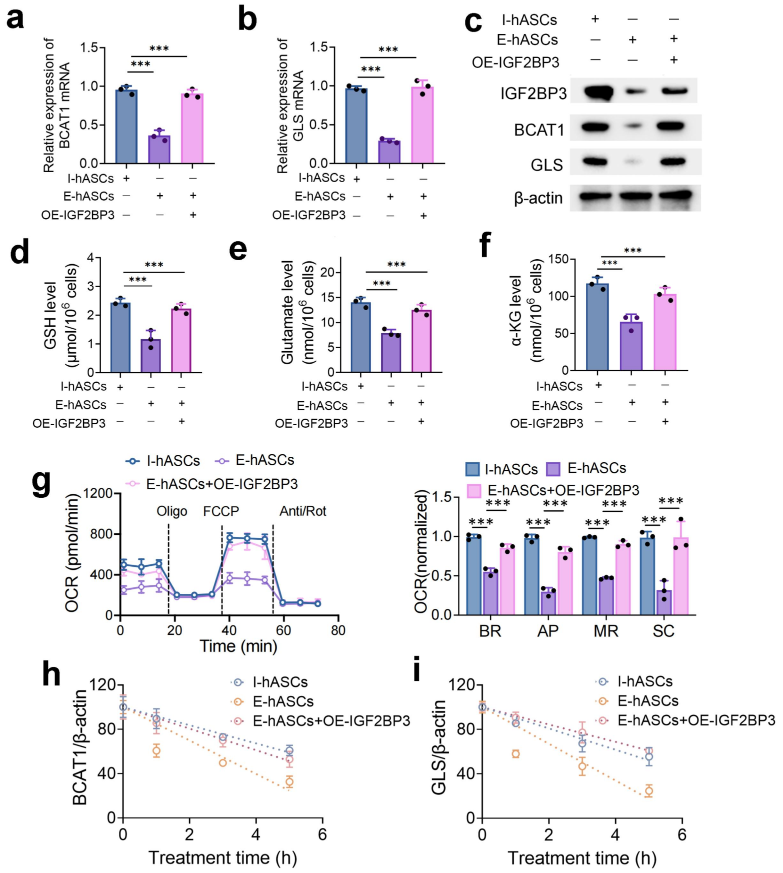

Supplementary Fig. S10. The crucial role of IGF2BP3 in restoring the metabolic homeostasis in E-hASCs.

a-b. Differential expression of BCAT1 (a) and GLS (b) mRNA among I-hASCs, E-hASCs and E-hASCs with IGF2BP3 overexpression detected by qRT-PCR. c. Western blotting was used to detect the expression of IGF2BP3, BCAT1, and GLS in I-hASCs, E-hASCs and E-hASCs with IGF2BP3 overexpression. d-f. Levels of GSH (d), Glutamate (e), and α-KG (f) in I-hASCs, E-hASCs and E-hASCs with IGF2BP3 overexpression. g. The OCRs of I-hASCs, E-hASCs and E-hASCs with IGF2BP3 overexpression, and BR, AP, MR, and SC quantifications in each group. h-i. Stability of BCAT1 (h) and GLS (i) mRNA in I-hASCs, E-hASCs and E-hASCs with IGF2BP3 overexpression. * * * represent *P*-value < 0.001.

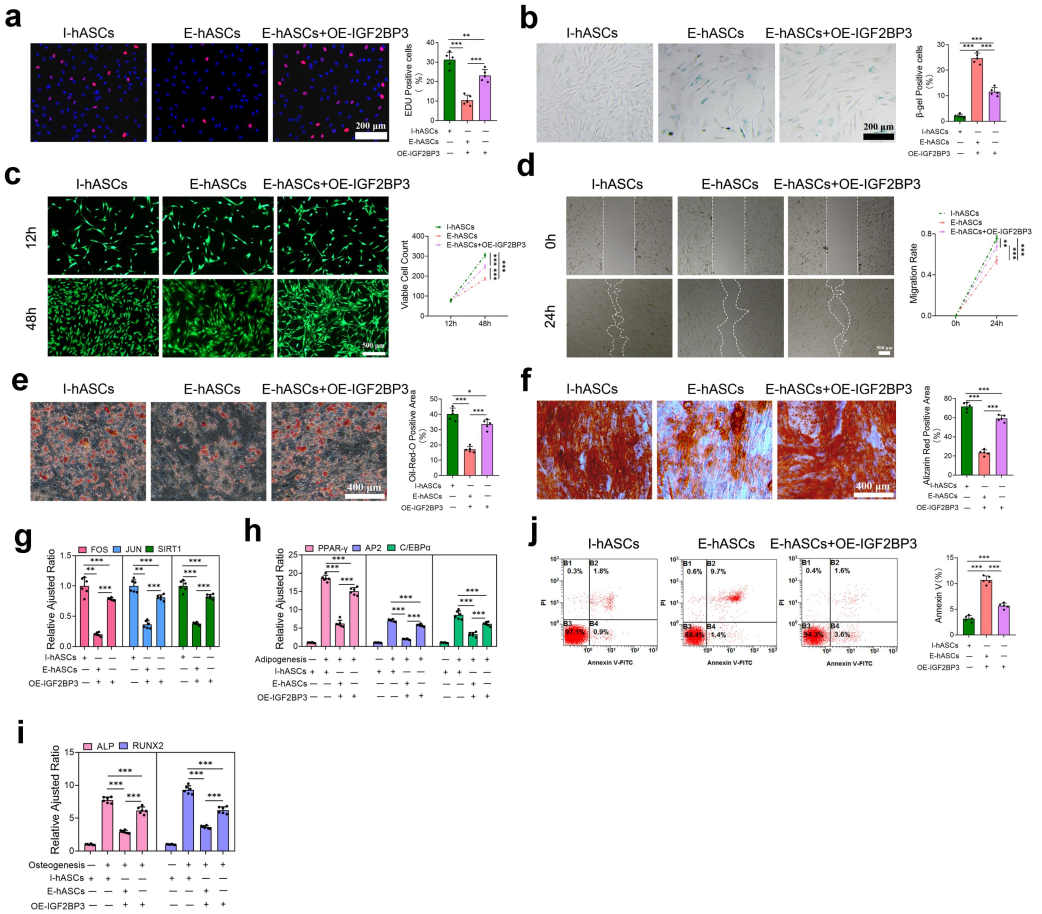

Supplementary Fig. S11. IGF2BP3 overexpression reverses the aging-associated cellular dysfunction of E-hASCs.

a. Representative images of EdU staining and quantification of the percentage of EdU-positive cells for I-hASCs, E-hASCs and E-hASCs with IGF2BP3 overexpression. b. SA-β-gal staining and quantification of the percentages of SA-β-gal-positive cells for I-hASCs, E-hASCs and E-hASCs with IGF2BP3 overexpression. c. Representative images of calcein-AM/PI staining and the quantification of viable cells after 12 and 48 h for I-hASCs, E-hASCs and E-hASCs with IGF2BP3 overexpression. d. Representative images of scratch assay and quantification of migration rates after 24 h for I-hASCs, E-hASCs and E-hASCs with IGF2BP3 overexpression. e. Oil Red O staining and quantification of the staining-positive areas for I-hASCs, E-hASCs and E-hASCs with IGF2BP3 overexpression after three weeks of adipogenic induction. f. Alizarin Red S staining and quantification of the staining-positive areas for I-hASCs, E-hASCs and E-hASCs with IGF2BP3 overexpression after three weeks of osteogenic induction. g. Relative expression of age-related genes in I-hASCs, E-hASCs and E-hASCs with IGF2BP3 overexpression. h. Relative expression of lipogenic genes in I-hASCs, E-hASCs and E-hASCs with IGF2BP3 overexpression after adipogenic induction. i. Relative expressions of osteogenic genes of I-hASCs, E-hASCs and E-hASCs with IGF2BP3 overexpression after osteogenic induction. j. Apoptosis analysis and the quantification of apoptotic cells for I-hASCs, E-hASCs and E-hASCs with IGF2BP3 overexpression. * represent P-value < 0.05; ** represent P-value < 0.01; * * * represent P-value < 0.001.

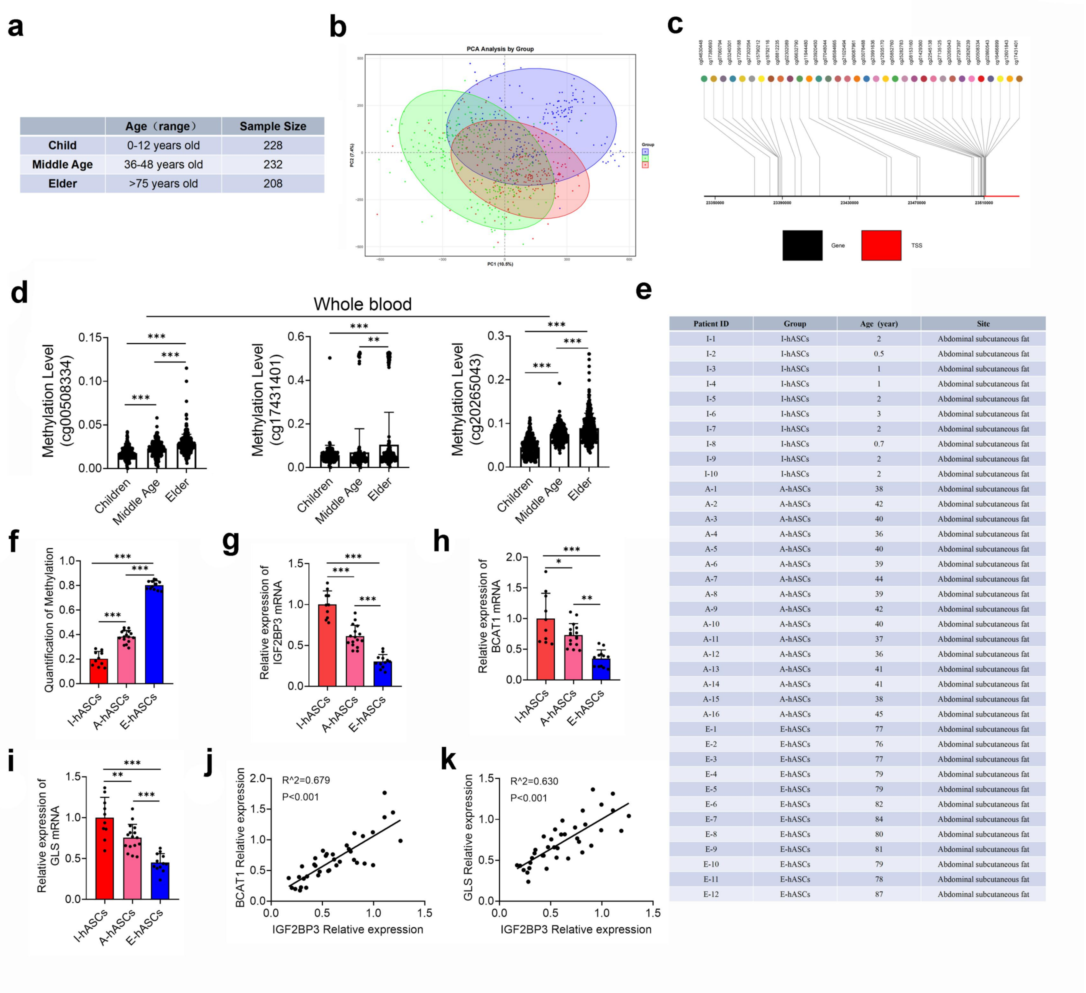

	Age（range）	Sample Size
Child	0-12 years old	228
Middle Age	36-48 years old	232
Elder	>75 years old	208

Patient ID	Group	Age (year)	Site
I-1	I-hASCs	2	Abdominal subcutaneous fat
I-2	I-hASCs	0.5	Abdominal subcutaneous fat
I-3	I-hASCs	1	Abdominal subcutaneous fat
I-4	I-hASCs	1	Abdominal subcutaneous fat
I-5	I-hASCs	2	Abdominal subcutaneous fat
I-6	I-hASCs	3	Abdominal subcutaneous fat
I-7	I-hASCs	2	Abdominal subcutaneous fat
I-8	I-hASCs	0.7	Abdominal subcutaneous fat
I-9	I-hASCs	2	Abdominal subcutaneous fat
I-10	I-hASCs	2	Abdominal subcutaneous fat
A-1	A-hASCs	38	Abdominal subcutaneous fat
A-2	A-hASCs	42	Abdominal subcutaneous fat
A-3	A-hASCs	40	Abdominal subcutaneous fat
A-4	A-hASCs	36	Abdominal subcutaneous fat
A-5	A-hASCs	40	Abdominal subcutaneous fat
A-6	A-hASCs	39	Abdominal subcutaneous fat
A-7	A-hASCs	44	Abdominal subcutaneous fat
A-8	A-hASCs	39	Abdominal subcutaneous fat
A-9	A-hASCs	42	Abdominal subcutaneous fat
A-10	A-hASCs	40	Abdominal subcutaneous fat
A-11	A-hASCs	37	Abdominal subcutaneous fat
A-12	A-hASCs	36	Abdominal subcutaneous fat
A-13	A-hASCs	41	Abdominal subcutaneous fat
A-14	A-hASCs	41	Abdominal subcutaneous fat
A-15	A-hASCs	38	Abdominal subcutaneous fat
A-16	A-hASCs	45	Abdominal subcutaneous fat
E-1	E-hASCs	77	Abdominal subcutaneous fat
E-2	E-hASCs	76	Abdominal subcutaneous fat
E-3	E-hASCs	77	Abdominal subcutaneous fat
E-4	E-hASCs	79	Abdominal subcutaneous fat
E-5	E-hASCs	79	Abdominal subcutaneous fat
E-6	E-hASCs	82	Abdominal subcutaneous fat
E-7	E-hASCs	84	Abdominal subcutaneous fat
E-8	E-hASCs	80	Abdominal subcutaneous fat
E-9	E-hASCs	81	Abdominal subcutaneous fat
E-10	E-hASCs	79	Abdominal subcutaneous fat
E-11	E-hASCs	78	Abdominal subcutaneous fat
E-12	E-hASCs	87	Abdominal subcutaneous fat

Supplementary Fig. S12. The regulation of up- and down-stream of IGF2BP3 in hASCs during aging axis.

a. The age groupings and smaple sizes for DNA methylation analyses conducted on whole blood samples obtained from the Illumina-450 K DNA methylation microarray in the GEO dataset. b. PCA presented the heterogeneity among Child (purple), Middle Age (pink) and Elder (green) groups. c. Graphic illustration of potential age-associated DNA methylation sites of the IGF2BP3 promoter in whole blood. d. The differences of methylation levels of the specific DNA methylation sites (cg00508334, cg17431401 and cg20265043) of the IGF2BP3 promoter in whole blood. e. The groupings of I-hASCs, A-hASCs, and E-hASCs, as well as the information of sample sources. f. Methylation-specific PCR (MSP) was used to detect the methylation status of the CpG islands in the IGF2BP3 core promoter region in I-hASCs (10 samples), A-hASCs (16 samples), and E-hASCs (12 samples). g-i. Differential expression of IGF2BP3 (g), BCAT1 (h) and GLS (i) mRNA among I-hASCs, A-hASCs and E-hASCs detected using qRT-PCR. j-k. Pearson correlations of IGF2BP3 and BCAT1 mRNA relative expression (j), and IGF2BP3 and GLS mRNA relative expression (k) in entire hASCs obtained from 38 samples. * represent *P*-value < 0.05;* * represent *P*-value < 0.01; * * * represent *P*-value < 0.001.

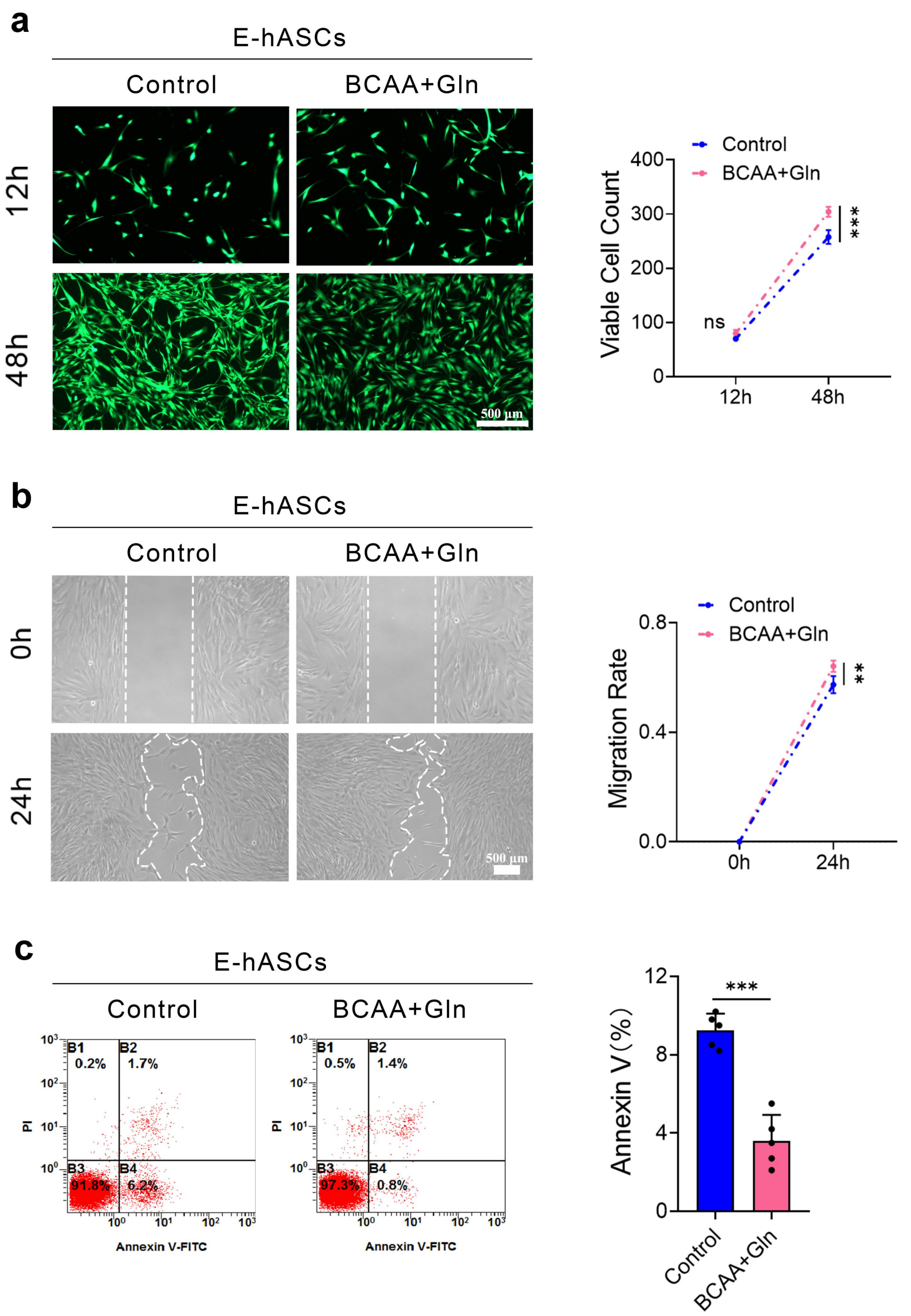

Supplementary Fig. S13. Supplementation with BCAA and glutamine reversed the cell viability of E-hASCs.

a. Representative images of calcein-AM/PI staining and quantification of viable cells after 12 and 48 h for E-hASCs after BCAA and Gln supplementation. b. Representative images of scratch assay and quantification of migration rates after 24 h for E-hASCs after BCAA and Gln supplementation. c. Apoptosis analysis and quantification of apoptotic cells in E-hASCs after BCAA and Gln supplementation. * * represent *P*-value < 0.01; * * * represent *P*-value <0.001.

a

Sample	① SV40	② HGAPDH	ΔCT (①-②)
ADHX-C1061#hTERT_ SV40-T#Poolcells	14.92±0.04	16.90±0.08	-1.98
ADHX-C1061	Undetermined	16.43±0.08	NA

b

Sample	① hTERT	② HGAPDH	ΔCT (①-②)
ADHX-C1061#hTERT_ SV40-T#Poolcells	20.36±0.12	16.90±0.08	3.46
ADHX-C1061	Undetermined	16.43±0.08	NA

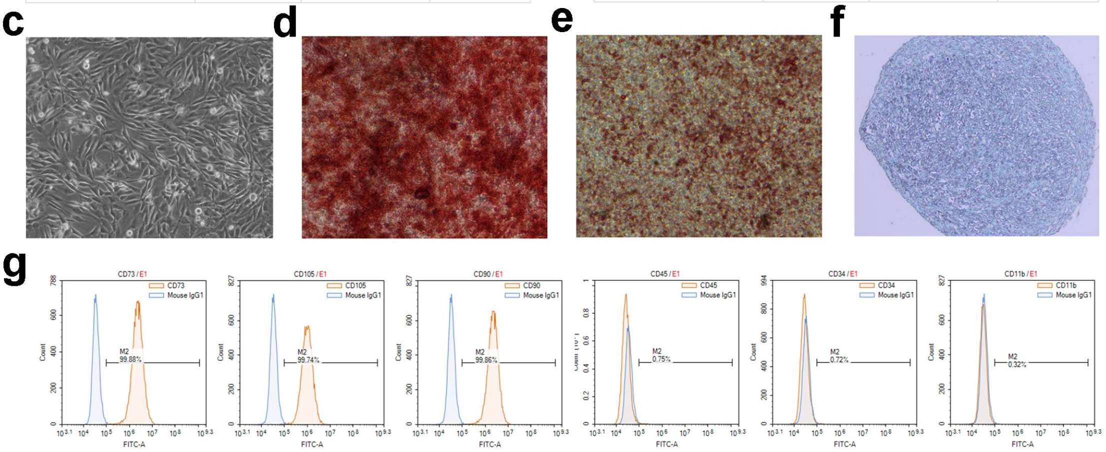

Supplementary Fig. S14. The quality inspection certificate of the immortalized hASCs cell line (ADHX-C1061) obtained from HyCyte™.

a-b. The expression levels of SV40 (a) and hTERT (b) mRNAs in ADHX-C1061 obtained from HyCyte™ (Suzhou, China). c. Representative images of cell morphology of ADHX-C1061 under a light microscope (×100). d-f. Representative images of osteogenic (d), adipogenic (e), and chondrogenic (f) differentiation of ADHX-C1061(×200). g. The cell surface marker expression of hASCs CD73, CD105, CD90, CD45, CD34, and CD11b, were evaluated by flow cytometry.

Supplementary Tables

Supplementary Table 1. Sample sources and group information.

Patient No.	Group	Sex	Age	BMI	Surgical reasons	Surgery type	Sites for obtaining fat
I1	I-hASCs	Male	2	14.8	Scar contracture after burn	Full-thickness skin graft	Abdominal subcutaneous fat
I2	I-hASCs	Female	3	15.1	Scar contracture after burn	Full-thickness skin graft	Abdominal subcutaneous fat
I3	I-hASCs	Female	1	15.5	Scar contracture after burn	Flap transplantation	Abdominal subcutaneous fat
I4	I-hASCs	Male	1	15.7	Maxillofacial traumatic injuries	Full-thickness skin graft	Abdominal subcutaneous fat
I5	I-hASCs	Male	1	14.6	Scar contracture electrocution injuries	Full-thickness skin graft	Abdominal subcutaneous fat
I6	I-hASCs	Female	3	16.2	Scar contracture after burn	Flap transplantation	Abdominal subcutaneous fat
E1	E-hASCs	Female	78	22.4	Scar contracture electrocution injuries	Full-thickness skin graft	Abdominal subcutaneous fat
E2	E-hASCs	Female	82	25	Pressure ulcers	Flap transplantation	Abdominal subcutaneous fat
E3	E-hASCs	Male	80	24.2	Scar contracture after burn	Flap transplantation	Abdominal subcutaneous fat
E4	E-hASCs	Male	82	26	Maxillofacial traumatic injuries	Full-thickness skin graft	Abdominal subcutaneous fat
E5	E-hASCs	Female	77	25.5	Scar contracture after burn	Flap transplantation	Abdominal subcutaneous fat
E6	E-hASCs	Male	83	23	Pressure ulcers	Flap transplantation	Abdominal subcutaneous fat

Supplementary Table 2. Primers used for qRT-PCR.

Gene	Forward(5'-3')	Reverse(5'-3')
IGF2BP3	ACGAAATATCCCGCCTCATTTAC	GCAGTTTCCGAGTCAGTGTTCA
BCAT1	AGCCCTGCTCTTTGTACTCTT	CCAGGCTCTTACATACTTGGGA
GLS	AGGGTCTGTTACCTAGCTTGG	ACGTTCGCAATCCTGTAGATTT
PPARγ	GACCACTCGCATTCCTTT	GAGTGGGTGTCGCTGTTGAA
C/EBPα	CTGATTCTTGCCAAACTGAG	GAGGAAGCTAAGACCCACTAC
ALP	ACGTGGCTAAGAATGTCATC	CTGGTAGGCGATGTCCTTA
AP2	AAATCACCGCAGACGACAG	TCGACTTTCCATCCCACTTC
RUX2	AACAGCAGCAGCAGCAGCAG	GCACCGAGCACTAGAAGTTGG
FOS	CCGGGGATAGCCTCTCTTACT	CCAGGTCCGTGCAGAAGTC
JUN	TCCAAGTGCCGAAAAAGGAAG	CGAGTTCTGAGCTTTCAAGGT
SIRT1	TAGCCTTGTCAGATAAGGAAGGA	ACAGCTTCACAGTCAACTTTGT
SV40	ATTTGCCTTCAGGTCAGGGA	TGCGTTCCAGGCAATGCTTTA
hTERT	TCCTCAGGAACACCAAGAAGTT	AAACGTGGTCTCCGTGACAT
GAPDH	GGGAAACTGTGGCGTGAT	GAGTGGGTGTCGCTGTTGAA

Supplementary Table 3. Primers used for m6A-RIP-qPCR.

m6A sites	Forward(5'-3')	Reverse(5'-3')
BCAT1 (+70)	GCTACGACCCTTGGGAT	GTAGCTGGTGTGACTAT
BCAT1 (+659)	CCAGTGTCCCTGTGGGCCAAT	GATCTGATGGTCCTCTCCAT
BCAT (+1221)	GAGGATACCAACTGTATGCTACT	CAATCACTCTTGTAACACATTGAT
BCAT (+7669)	GTAGAAGCACAATTAAACATC	GAATGTGTCTATTGCCAGCA
BCAT (+8438)	GAAGTACAGCAAAACCTG	CACAGGTTGACAGGGTCTGGAG
GLS (+904)	GCAGATTATATTCCTCAACT	CCAACATATCGATGCACATA
GLS (+945)	GTTGGAAAAGAGCCGAGT	CTGCATGACATAGTCAAATT
GLS (+1748)	GCTGCATATACTGGAGATG	CATGACCCTCTGCAGCAGCT
GLS (+3378)	GTGGCATGTTAGTGAGGAGT	GCTGTATGTGATAACAACTAA
GLS (+4376)	AGCTGCAGAGCTGGATTAGCT	TACATTCATAGCTGTGTAATCC

Supplementary Table 4. shRNA sequences of target genes.

Gene	shRNA#1(5'-3')	shRNA#2(5'-3')
IGF2BP3	CGGTGAATGAACTTCAGAATT	GCTGAGAAGTCGATTACTA
METTL3	CCAGTCATAAACCAGATGAAA	CGTCAGTATCTTGGGCAAGTT
BCAT1	GCCCAATGTGAAGCAGTAGATA	GCCGCATCTTGAGCAAATT
GLS	AACGTTTCAGTCTGAAAGAGAA	CACGATCTTGTTTCTCTGTGTA

Supplementary Table 5. The primer sequences used for the MSP assay.

**IGF2BP3 promoter (Methylated)**	Forward: 5’- GTAATTAATGGTAAACGCGAAGTC -3’
	Reverse: 5’- CGAAATAATAAAAAATAAAATCCCG -3’
**Loading control**	Forward: 5’- TAATTAATGGTAAATGTGAAGTTGA -3’
	Reverse: 5’- CAAAATAATAAAAAATAAAATCCCACA -3’

Supplementary Materials and Methods

The scRNA-seq data processing, dimensional reduction, and clustering

The raw scRNA-seq data were deposited in the NCBI Gene Expression Omnibus (GEO) under accession number GSE 267783 and processed using an internal pipeline to create gene expression matrices. Raw sequencing reads were subjected to quality assessment using FastQC (version 0.11.7) and Fastp to remove low-quality reads[1,2], followed by adapter sequence removal using cutadapt[3]. After extracting the cell barcode and UMI, sequencing reads were mapped to the GRCh38 reference genome (Ensembl version 92 annotation) using the STAR software (version 2.5.3a) [4]. FeatureCounts (version 1.6.2) software was employed to calculate the UMI and gene counts per cell [5]. Cells with gene count < 200 in the top 5%, UMI counts in the top 5%, and > 50% mitochondrial content were excluded. Seurat (version 3.1.2) was used to facilitate data normalization, dimension reduction, clustering, and differential expression analysis[6]. The top 2,000 genes with the greatest variability were selected for PCA, and cells were clustered using FindClusters based on the top 20 principal components. Then, the UMAP algorithm was performed to visualize cells in different dimensions [7].

Cell-cell interaction analysis in scRNA-seq

CellphoneDB was used to forecast cell-cell interactions using established ligand-receptor pairs [8]. The number of permutations used to calculate the null distribution of the average ligand-receptor pair expression with randomized cell identities was set to 1,000. The ligand or receptor expression threshold was determined based on the average log of the gene expression distribution across all cell clusters. Predicted ligand-receptor interaction pairs with P-value < 0.05 and average log expression > 0.1 were deemed statistically significant and visualized using heatmap plots in CellphoneDB.

TF regulatory network analysis

A TF gene regulatory network was developed using PySCENIC (version 0.11.0) with a single-cell RNA expression matrix and TFs in AnimalTFDB [9]. Initially, GRNBoost2 was used to predict a co-expression network to identify potential targets for TFs. Subsequently, the i-cistarget software was used to filter indirect targets and identify TF-binding motifs. Afterward, regulon activity in each cell was scored using AUCell. Cell cluster-specific TF regulons were identified according to the Regulon Specificity Scores, and heatmap plots were used to visualize TF regulon activity.

Pseudotime analysis

RNA velocity analysis was conducted using BAM files of hASCs aligned to the GRCh38/hg38 reference genome. The analysis was performed with velocyto (v.17.17) and scVelo (v.2.3) in Python, utilizing default parameters [10]. The results were visualized on a UMAP plot generated from Seurat clustering analysis to ensure consistency in visualization. Additionally, the PAGA algorithm was applied by constructing a symmetrized kNN-like graph based on PCA data using the scanpy.tl.paga function with default settings, incorporating approximate nearest neighbor search within UMAP [11]. The PAGA graph was generated for each partitioning to demonstrate the connectivity and pseudotime ordering of hASCs differentiation. Moreover, the values ranging from 0 to 1 in the latent time featureplot represented the progression from early to late time points in cell generation, with indicating early and 1 indicating late stages.

Cell developmental potential evaluation

In order to assess the developmental potentials and stemness of hASCs subpopulations, the CytoTRACE score was calculated using the R package CytoTRACE v0.3.3. The CytoTRACE algorithm was used to predict the differentiation state of hASCs from scRNA-Seq data using gene counts and expression[12]. CytoTRACE scores range from 0 to 1, while higher scores indicate higher stemness and vice versa.

UCell gene set scoring

The UCell method was employed to evaluate pathway enrichment across different cell clusters. UCell scores were calculated utilizing the Mann-Whitney U statistic, which ranks query genes based on their expression levels within individual cells[13]. Gene set scoring was conducted through the utilization of the UCell v 1.1. R package.

Liquid chromatography-mass spectrometry/mass spectrometry (LC-MS/MS) analysis

High-resolution LC-MS/MS analysis was performed using a Q-Exactive mass spectrometer (Thermo Scientific, United States) with an Easy-nLC system (Thermo Scientific, United States). The peptides were loaded onto a reverse-phase C18 trap column (Thermo Scientific, United States) equilibrated with solvent A (2% acetonitrile, 0.1% formic acid) and solvent B (84% acetonitrile, 0.1% formic acid) at a flow rate of 300 nL/min, controlled using IntelliFlow technology. The Q Exactive HF used a data-dependent acquisition method for automatic switching between full-scan MS and MS/MS acquisition. The normalized collision energy was set to 30 eV. Mass spectra were acquired in full-scan mode over a range of 400–2000 m/z with a resolution of 60,000, and the 15 most abundant precursor ions from the survey scan were used for energy collisional dissociation (HCD) fragmentation. The automatic gain control target was 3e6, with a maximum injection time of 50 ms. Dynamic exclusion with an exclusion time of 15 s was implemented to acquire survey scans at a resolution of 70,000 at m/z 200, while the resolution of the HCD spectra was set to 17,500 at m/z 200.

Identification of proteins

The raw mass spectrometry (MS) proteomics data were submitted to the ProteomeXchange Consortium (http://proteomecentral.proteomexchange.org) via the iProX partner repository and assigned a PXD number: PXD051832. The data files for each sample were processed with MaxQuant software (version 1.6.5.0). The MASCOT server (version 2.5.1) was used to search for peak lists against the UniProt and SwissProt databases of human proteins. The search criteria were as follows: trypsin/P was chosen as the enzyme with two missed cleavages allowed; carbamidomethylation on cysteine was set as a fixed modification and oxidation and N-term acetylation as a variable modification; peptide mass tolerance was set at ± 20 ppm, and mass tolerance of fragment was ± 20 ppm, with a 1% false discovery rate. At least one unique peptide was identified to support protein identification. The consistency analysis of the 12 samples was performed using PCA and a sample repeatability heatmap plot.

Identifying differentially expressed genes (DEGs) and proteins (DEPs)

DEGs were identified using the FindMarkers function in Seurat during scRNA-seq analysis.

Genes expressed in at least 10% of the cells in one cluster with an average log(Fold Change) value greater than 0.25 and P-value < 0.05 were identified as DEGs using the Wilcoxon Rank Sum test with default settings. Manual annotation of cell types for each cluster was performed using canonical marker expression and literature insights, with dot plots illustrating the marker expression for each cell cluster. Markers were excluded when identified as markers for different cell clusters. Pearson correlation analysis of DEGs in scRNA-seq was performed using the R package cor.test.

The DEPs in bulk hASCs between infants and elders were identified using a t-test (P-value < 0.05) and fold changes > 1.2 or < 0.83, and the results were visualized as a volcano plot. The overlaps between DEGs and DEPs detected in I-hASCs and E-hASCs at a bulk level were assessed and visualized using Venn diagrams (http://bioinformatics.psb.ugent.be/webtools/Venn/). PRM analysis and the targeted protein quantification were performed to verify DEPs.

Pathway enrichment analysis

To investigate the potential functions of DEGs and DEPs in primary hASCs, GO-BP and Kyoto encyclopedia of genes and genomes (KEGG) enrichment analyses were performed using the "clusterProfiler" R package (version 3.16.0) [14]. According to DEPs, the enriched metabolic pathways between I-hASCs and E-hASCs were demonstrated using a heatmap plot. The Sankey bubble diagram and chord plot presented the highly expressed DEPs of I-hASCs and related enriched metabolic pathways, as well as DEGs highly expressed in cluster 1 of hASCs and related enriched metabolic pathways. GSEA was performed using GSEA software (version 3.0) to identify highly enriched metabolic pathways in cluster 1 compared to the other hASC cell clusters.

Data processing of targeted metabolomics analysis

MultiQuant (version 3.0.3) was applied to process data. Quality control samples were used to ensure the stability and repeatability of the metabolomics method. Metabolites were considered reproducible if their coefficients of variation were < 30%. After normalizing the peak intensity, the processed data were uploaded before being imported into SIMCA-P (version 14.1; Umetrics, Sweden) for multivariate data analysis. The variable importance in projection was calculated using an orthogonal partial least squares discriminant analysis model. Statistical significance was assessed using an unpaired Student's t-test, with a threshold of P-value < 0.05.

DNA methylation analysis

The DNA methylation analysis of the whole blood was performed using Illumina's 450 K DNA methylation microarray sourced from GSE102177, GSE104812, GSE106648, GSE54643, GSE55763, GSE59065, GSE59507, GSE59685, GSE113725, GSE60132, GSE61151, GSE64491, GSE64495, GSE67444, GSE67490, GSE107143, GSE111629, GSE116378, GSE116379, GSE120307, GSE32148, GSE40279, GSE43414, GSE67530, GSE67705, GSE67751, GSE52588, GSE53128, GSE53740, GSE53840, GSE72680, GSE72773, GSE72774, GSE72775, GSE72776, GSE72777, GSE80283, GSE82273, GSE83334, GSE84727, GSE85506, GSE87571, GSE87648, GSE95049, GSE97362, and GSE9986 in GEO database. Initially, we analyzed the whole-blood DNA methylation profiles of healthy individuals from the aforementioned GSE datasets. We then isolated the methylation data corresponding to three age categories (Children: 0-12 years old,

Middle Age: 36-48 years old, Elder: > 75 years old) for subsequent investigation. Gaussian Mixture Quantile Normalization (GMQN) was applied for batch effect correction, and (K-nearest neighbor) KNN method was used to fill in missing values [15]. Beides, data normalization was performed with β-mixture quantile (BMIQ) method[16]. The differentially methylated positions (DMPs) among multiple groups were assessed using one-way analysis of variance (ANOVA) , employing a significance threshold of adjusted P-value < 0.05 and absolute deltaBeta > 0.20. Methylation status of IGF2BP3 promoter was further evaluated via Methylation Specific PCR (MSP) as previously discribed [17]. The primer sequences used for the MSP assay are presented in Supplementary Table 5.

References

1 Andrews, S. Babraham Bioinformatics -FastQC A Quality Control tool for High Throughput Sequence Data. (2013).

2 Chen, S., Zhou, Y., Chen, Y. & Gu, J. J. C. S. H. L. fastp : an ultra-fast all-in-one FASTQ preprocessor. (2018).

3 Karlsson, F. H. *et al.* Symptomatic atherosclerosis is associated with an altered gut metagenome. *Nature communications* **3**, 1245, doi:10.1038/ncomms2266 (2012).

4 Dobin, A. *et al.* STAR: ultrafast universal RNA-seq aligner. *Bioinformatics (Oxford, England)* **29**, 15-21, doi:10.1093/bioinformatics/bts635 (2013).

5 Liao, Y., Smyth, G. K. & Shi, W. featureCounts: an efficient general purpose program for assigning sequence reads to genomic features. *Bioinformatics (Oxford, England)* **30**, 923-930, doi:10.1093/bioinformatics/btt656 (2014).

6 Wang, Q. *et al.* Single-cell profiling guided combinatorial immunotherapy for fast-evolving CDK4/6 inhibitor-resistant HER2-positive breast cancer. *Nature communications* **10**, 3817, doi:10.1038/s41467-019-11729-1 (2019).

7 Turner, T. C. *et al.* Harnessing lipid signaling pathways to target specialized pro-angiogenic neutrophil subsets for regenerative immunotherapy. *Science advances* **6**, doi:10.1126/sciadv.aba7702 (2020).

8 Efremova, M., Vento-Tormo, M., Teichmann, S. A. & Vento-Tormo, R. CellPhoneDB: inferring cell-cell communication from combined expression of multi-subunit ligand-receptor complexes. *Nature protocols* **15**, 1484-1506, doi:10.1038/s41596-020-0292-x (2020).

9 Van de Sande, B. *et al.* A scalable SCENIC workflow for single-cell gene regulatory network analysis. *Nature protocols* **15**, 2247-2276, doi:10.1038/s41596-020-0336-2 (2020).

10 La Manno, G. *et al.* RNA velocity of single cells. *Nature* **560**, 494-498, doi:10.1038/s41586-018-0414-6 (2018).

11 Wolf, F. A. *et al.* PAGA: graph abstraction reconciles clustering with trajectory inference through a topology preserving map of single cells. *Genome biology* **20**, 59, doi:10.1186/s13059-019-1663-x (2019).

12 Gulati, G. S. *et al.* Single-cell transcriptional diversity is a hallmark of developmental potential. *Science (New York, N.Y.)* **367**, 405-411, doi:10.1126/science.aax0249 (2020).

13 Andreatta, M. & Carmona, S. J. UCell: Robust and scalable single-cell gene signature scoring. *Computational and structural biotechnology journal* **19**, 3796-3798, doi:10.1016/j.csbj.2021.06.043 (2021).

14 Yu, G., Wang, L. G., Han, Y. & He, Q. Y. clusterProfiler: an R package for comparing

biological themes among gene clusters. *Omics : a journal of integrative biology* **16**, 284-287, doi:10.1089/omi.2011.0118 (2012).

15 Moindjie, H. *et al.* Preimplantation factor is an anti-apoptotic effector in human trophoblasts involving p53 signaling pathway. *Cell death & disease* **7**, e2504, doi:10.1038/cddis.2016.382 (2016).

16 Lapatto, H. A. K. *et al.* Nicotinamide riboside improves muscle mitochondrial biogenesis, satellite cell differentiation, and gut microbiota in a twin study. *Science advances* **9**, eadd5163, doi:10.1126/sciadv.add5163 (2023).

17 Wu, P. *et al.* Metformin Suppresses Hypopharyngeal Cancer Growth by Epigenetically Silencing Long Non-coding RNA SNHG7 in FaDu Cells. *Frontiers in pharmacology* **10**, 143, doi:10.3389/fphar.2019.00143 (2019).
