# Supplementary material for: IGF2BP3-dependent glutamine/BCAA metabolic rewiring rejuvenates aged human adipose-derived stem cells for enhanced tissue regeneration
Source: Cell Discov. 2026 Jan 20;12:5. doi: 10.1038/s41421-025-00860-7 (PMC12819398; doi:10.1038/s41421-025-00860-7)
Supplement: Supplementary file 2 — Informed Consent [file 41421_2025_860_MOESM2_ESM.pdf]

知情同意书

项目名称：深、浅筋膜来源的自体腹部脂肪移植对烧伤后瘢痕治疗效果的对照研究

组长单位：西京医院烧伤与皮肤外科

项目负责人：王洪涛

研究年限：2021.01.01 日—2024.07.31 日

版本号：V2.0

患者姓名：[redacted]

患者 ID：[redacted]

尊敬的参与者：

您将被邀请参加一项“ 深、浅筋膜来源的自体腹部脂肪移植对烧伤后瘢痕治疗效果的对照研究 ”。该研究由西京医院烧伤与皮肤外科王洪涛教授牵头开展，研究将严格遵从《赫尔辛基宣言》和我国相关的法律法规要求。

在您决定是否参加该项临床研究之前，请仔细阅读以下内容，它可以帮助您充分了解为何要进行该项研究目的、研究程序和参加研究后可能给您带来的益处、风险和不适。

一、研究目的

研究不同层次、年龄来源的腹部脂肪及脂肪干细胞对于创面修复和瘢痕效果的比较，为后期创面.瘢痕治疗提供新的思路和选择

二、研究方案

本研究是一项临床前研究，分别获取不同层次以及不同年龄来源吸脂病人、需要游离腹部全层皮片、腹部皮瓣的病人所丢弃废用的脂肪用于动物实验或脂肪干细胞的提取。

一方面将获得的脂肪解离为血管基质细胞（SVF）和脂肪干细胞用于单细胞测序和后续的细胞实验，从而揭示不同层次以及不同年龄来源的 SVF 和脂肪干细胞的组成异质性；另一方面将收集到的脂肪不同层次以及不同年龄的颗粒脂肪和脂肪干细胞作用于创面和烧伤后的瘢痕，探究其在组织再生领域，对创面愈合和瘢痕防治的作用效果。

四、研究过程

入排标准：

4.3.1 入选标准：1）0 岁-90 岁非肿瘤和非全身感染的病人；2）腹部吸脂病人：3）需要游离腹部全层皮片和进行腹部皮瓣移植的病人；4）患者完全理解本研究的研究目的和方案；4）患者自愿提供废弃脂肪，并签署知情同意书。

4.3.2 排除标准：：1）肿瘤和全身感染的病人；2）处于疾病终末期可能失访的病人；3）腹部脂肪取样位置皮下局部感染、纤维化或其他病变；4）存在会影响皮肤及皮下脂肪生理状态的良恶性疾病，如神经纤维瘤，银屑病等；5）不能完

全理解本研究目的、方案，不能积极配合获取皮下脂肪标本的患者；6）孕妇及哺乳期女性。

五、风险与不适

本研究获取的是吸脂病人、需要游离腹部全层皮片、腹部皮瓣的病人所丢弃废用的脂肪，因此对病人无附加的风险和不适。

六、参加研究可能的受益

可获得更为全面的术后随访，提供更多的治疗指导和帮助。

七、个人信息保密

本研究中收集到的信息将作为机密保存。为了保护您的身份，在研究文件中任何关于您的信息都将使用统一格式的编号，而不是使用您的姓名。所收集和合并的所有受试者信息中，任何可以帮助识别您的身份的信息都将被去除，保证有关信息不能链接到一个特定的研究受试者。

伦理委员会和管理当局在不违反适用法律和法规所准许的范围内，在不侵犯受试者隐私的情况下，直接查阅受试者的原始医疗记录以便核查临床试验程序和/或数据（申办方不可以查阅），您或您合法代理人在签署知情同意书时即授权这种查阅。在适用法律和/或法规准许范围内，有关识别您的记录将保密并且不会公开，本研究的结果可能在医学杂志中发表或出于科学目的进行分享或由申办者用于产品研究或改进，但您的身份和个人信息在任何时候都将不会被披露。

八、退出研究

研究过程中研究医生会考虑您利益最大化，如果觉得您不适合在术中提供废弃脂肪标本，或者申办方/伦理委员会/国家政府部分要求停止，研究医生会向您主动解释原因并中止取样。您参加本次研究完全自愿，您有权选择不参加本次研究，也有权随时退出而不会因此受到处罚或利益损失。如果您准备退出研究，请及时告诉您的研究者。研究者得到可能影响您继续参加试验的信息，会及时通报您或您的监护人。

九、联系人及联系方式

您可随时了解与本研究有关的信息资料和研究进展，若发生与本研究相关的安全性新信息，我们也会及时通知您。如果您有与本研究有关的问题，或您在研

究过程中发生了任何不适与损伤，或有关于本项研究参加者权益方面的问题您可以通过（电话号码：19981462601 ）与（联络员：李子超 ）联系。

十、伦理委员会联系方式

如果您对参与本研究的权益和健康有任何问题或诉求，您可以联系西京医院伦理委员会，联系电话： 029-84771794。

知情同意签字页

我已详细阅读了以上知情同意书，并理解了研究的目的以及参加研究的可能受益和风险。研究者已将以上医学术语作了明确解释。我有机会提出问题并且所有问题均得到了通俗易懂的答复。我可以选择不参加本项研究，或在任何时候通知负责医生后退出，我的任何医疗待遇与权益不会因此而受到影响。如果我需要其他治疗，或者我没有遵守研究计划，或者发生了与研究相关的损伤，或者有任何其他原因，负责医生可以终止我继续参与本项研究。

我已阅读了以上知情同意书并获得副本，我的医师也向我作了详尽的说明。我自愿参加本次临床试验。我同意有关方面对照我的原始医疗记录，检查核对试验研究收集的资料。

受试者正楷姓名：[redacted]　　患者电话：[redacted]

受试者签名：[redacted]　　日期：2021.05.17　（年/月/日/）

（注：受试者无民事行为能力时，需监护人签名；受试者为限制民事行为能力时，受试者及其监护人需要签名）

受试者正楷姓名：[redacted]　　与受试者关系：[redacted]

监护人签名：[redacted]　　监护人电话：[redacted]

日期：2021.05.17　（年/月/日）

公正见证人签名（如适用）：________　日期：________　（年/月/日）

（如果受试者或其监护人无阅读能力时，要求有一位公正见证人签字，公正见证人阅读知情同意书和其他书面材料，并见证知情同意。）

我确认已向病人详尽解释了本临床试验的有关内容，包括病人可能的获益和风险，并解答了患者提出的所有问题。

研究者签名（正楷）：[redacted]　　日期：2021.05.17　（年/月/日）

研究者电话：[redacted]　　日期：2021.05.17　（年/月/日）

版本号：V2.0
版本日期：2020.12.25
